# Modulating Phase Behavior in Fatty Acid-Modified Elastin-like Polypeptides (FAMEs): Insights into the Impact of Lipid Length on Thermodynamics and Kinetics of Phase Separation

**DOI:** 10.1021/jacs.3c12791

**Published:** 2024-02-14

**Authors:** Zhe Zhang, Christopher J. Lynch, Ying Huo, Somya Chakraborty, Paul S. Cremer, Davoud Mozhdehi

**Affiliations:** † Department of Chemistry, Syracuse University, Syracuse, New York 13244, United States; ‡ Department of Chemistry, Pennsylvania State University, University Park, Pennsylvania 16802, United States; § Fayetteville-Manlius High School, Manlius, New York 13104, United States; ∥ BioInspired Syracuse: Institute for Material and Living Systems, Syracuse University, Syracuse, New York 13244, United States

## Abstract

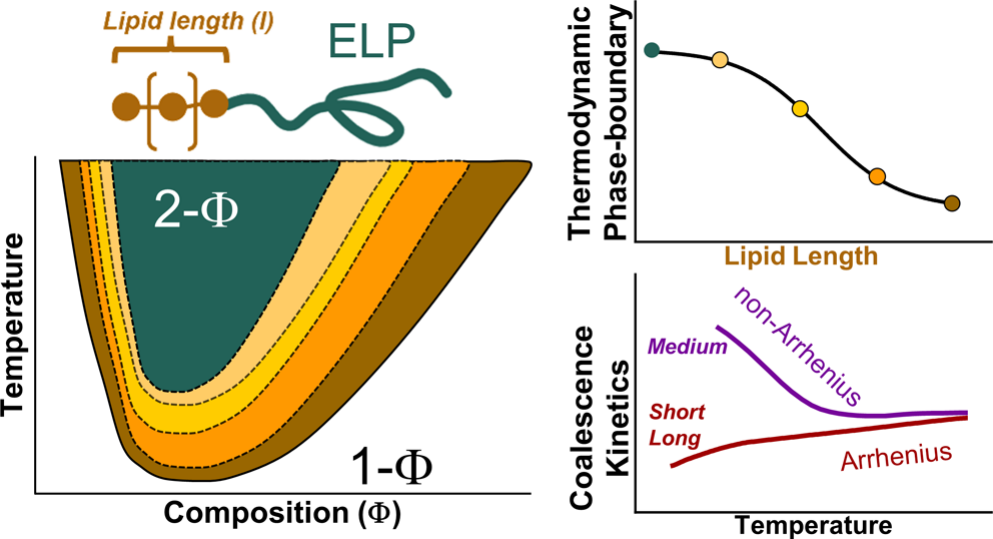

Although post-translational lipidation is prevalent in eukaryotes, its impact on the liquid–liquid phase separation of disordered proteins is still poorly understood. Here, we examined the thermodynamic phase boundaries and kinetics of aqueous two-phase system (ATPS) formation for a library of elastin-like polypeptides modified with saturated fatty acids of different chain lengths. By systematically altering the physicochemical properties of the attached lipids, we were able to correlate the molecular properties of lipids to changes in the thermodynamic phase boundaries and the kinetic stability of droplets formed by these proteins. We discovered that increasing the chain length lowers the phase separation temperature in a sigmoidal manner due to alterations in the unfavorable interactions between protein and water and changes in the entropy of phase separation. Our kinetic studies unveiled remarkable sensitivity to lipid length, which we propose is due to the temperature-dependent interactions between lipids and the protein. Strikingly, we found that the addition of just a single methylene group is sufficient to allow tuning of these interactions as a function of temperature, with proteins modified with C7–C9 lipids exhibiting non-Arrhenius dependence in their phase separation, a behavior that is absent for both shorter and longer fatty acids. This work advances our theoretical understanding of protein–lipid interactions and opens avenues for the rational design of lipidated proteins in biomedical paradigms, where precise control over the phase separation is pivotal.

## Introduction

Cells use membraneless organelles to regulate the spatiotemporal flow of life-sustaining matter, energy, and information.^1−4^ These systems are all-aqueous emulsions formed via liquid–liquid phase separation (LLPS) of biomacromolecules.^5^ Compared to synthetic water-in-water emulsions,^6−10^ biological condensates exhibit a far greater degree of structural complexity and functional tunability.^11^ For instance, cells regulate the formation and properties of their biological condensates through post-translational modifications (PTMs),^12,13^ achieving a level of precision that remains unmatched by synthetic systems.^14^ PTMs change the physicochemistry of modified amino acids and selectively “turn on/off” multivalent interactions that regulate the formation and stability of complex multiphase condensates.^15^ Matching nature’s mastery of PTMs to manipulate all-aqueous interfaces would confer better control over the formation, material properties, and performance of protein condensates, potentially paving the way to use this class of materials to replace traditional oil-based emulsions in food processing,^16^ cosmetics,^17^ biosensing,^18^ enrichment and purification of biologics,^19^ artificial cell design,^20,21^ and other biomedical and emerging applications.

With this inspiration, recent studies are taking cues from nature by applying PTMs to modulate the properties of condensates.^22−24^ The physicochemical diversity of post-translation modifications remains a rich and underutilized design parameter that is orthogonal to changing the amino acid sequence of proteins. However, little is known about how PTMs can alter the properties of proteins, regulate intricate chain configurations, and mediate dynamic multivalent interactions through intermolecular forces (e.g., hydrogen bonds, cation−π, ionic−π, etc.) that determine the properties of condensates. Currently, we lack a sufficiently detailed understanding of the physicochemical, structural, and dynamic factors to predictively correlate the observed condensate properties with protein sequences or their post-translational modification patterns.

Of the more than 300 types of post-translation modifications identified to date, lipidation is of particular interest because of its critical role in modulating protein function, localization, and interactions within cell membranes.^25^ Despite its prevalence in biology (and in phase-separating biological condensates), a systematic investigation of how lipidation influences the phase behavior of proteins is lacking.^26^ Although it is well established that the type of attached lipid determines biological outcomes, such as cell signaling and apoptosis,^27^ our molecular-level understanding of how these modifications influence protein properties in both solution and condensed phases remains incomplete. This knowledge gap exists because the diverse physicochemical properties of lipids attached to different proteins within cells present a potential challenge for systematic studies. For example, the various classes of lipids (e.g., fatty acids and prenols) can be physicochemically dissimilar and added to distinct locations (N- or C-termini) in different proteins. These complexities hinder the elucidation of robust transferable structure–property relationships.

**Figure 1:**
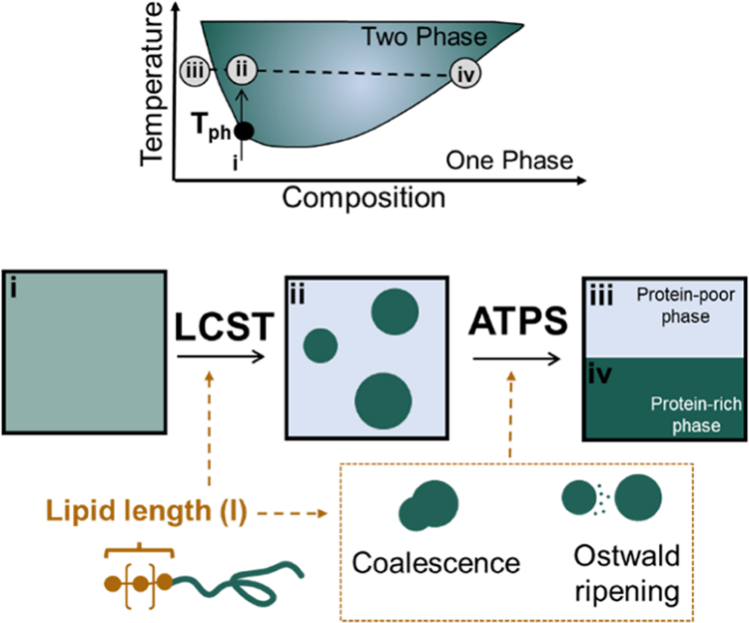
Schematic phase diagram of an ELP as a model protein with LCST. A solution of ELP (i) phase-separates as the temperature is increased above its phase separation temperature (*T*_ph_) to form protein-rich droplets (condensates) in solution (ii). This emulsion undergoes ATPS to form two phases with different protein compositions (iii, iv). Lipidation can alter both the thermodynamic phase boundary (LCST) and the kinetics of ATPS.

We therefore pursued a systematic investigation into how lipidation influences the phase behavior of a model protein, elastin-like polypeptides (ELPs).^28−31^ This intrinsically disordered protein, whose canonical sequence is (Gly–Xaa–Gly–Val–Pro), displays lower critical solution temperature (LCST) phase behavior. Above the binodal line, ELPs undergo LLPS and form phase-separated protein-rich droplets that eventually undergo ATPS formation. Two distinct phases are formed: a protein-rich (condensed phase) and a protein-poor phase (supernatant), as shown in Figure 1.

The thermodynamics and kinetics of ELP phase separation have been previously studied, establishing a baseline to quantify the effect of lipid molecular properties on the LCST phase boundary and ATPS formation. Notably, the phase separation temperature (*T*_ph_) of ELPs is inversely correlated to their hydrophobicity.^32−34^ Despite this intuitive relationship, a quantitative understanding of how hydrophobic motifs, such as lipids, modify the phase boundaries of ELPs is lacking. Most previous studies did not systematically control the number or physicochemical properties of attached groups, resulting in a mixture of products with varying hydrophobic molecular attachments.^35−37^ Counterintuitively, the addition of surfactants (e.g., sodium dodecyl sulfate) to ELP solutions does not significantly alter *T*_ph_ but influences the kinetics of ATPS formation by reducing the rate of droplet coalescence in favor of a competing mechanism, Ostwald ripening.^38^

In this study, we focused on saturated fatty acids as model PTMs for two reasons: (1) fatty acids represent a diverse and ubiquitous class of post-translational modifications in biological settings. Unlike other classes of lipidation that occur only on specialized proteins (e.g., cholesterol modification is only found on the hedgehog family of proteins), modification with fatty acids of different lengths is ubiquitously found in diverse classes of proteins in nature.^39^ These include acylation with short-, medium-, and long-chain fatty acids such as C2, C3, C4, C8, C14, and C16.^40^ (2) They also allow us to use a simple molecular variable, the number of carbons (lipid length, *l*), as a molecular dial to systematically vary the physicochemical properties of the attached lipids and to correlate changes in the phase boundary and stability of condensates with the molecular properties of lipids.

We hypothesized that lipid length alters the formation (thermodynamic phase boundaries) and kinetic stability of droplets formed by fatty acid-modified elastin-like polypeptides (FAMEs). Informed by our previous work,^41^ we suspected that lipid length modulates the thermodynamic phase boundaries via increasing unfavorable interactions between proteins and water and can alter the entropy of phase separation by micelle formation. We also envisioned that the fatty acid length could modulate the rate of ATPS by influencing both the Ostwald ripening and coalescence pathways. Specifically, increasing the lipid length should reduce the rate of the Ostwald ripening due to the decreased solubility of the molecules (unlike the effect of small-molecule surfactants). And because FAMEs possess amphiphilic characteristics and share molecular similarities with polymeric surfactants, lipid length could also reduce the rate of coalescence by stabilizing the protein droplets.

To test our hypothesis, we synthesized and characterized FAMEs by systematically altering the lipid length from C2 to C16 while maintaining a constant polypeptide length and polypeptide chemistry. Our aim was to quantitatively understand how lipid characteristics affect emulsions formed by phase-separating lipidated proteins. We find that the thermodynamic phase boundaries of FAMEs decrease sigmoidally as the lipid length increases. This suggests that a minimum length of the lipid is needed to induce the assembly of proteins, which can alter the entropy of phase separation. We also document that all FAMEs undergo ATPS formation via the coalescence mechanism, but lipid physicochemistry clearly alters the temperature dependence of ATPS formation rates. These observations indicate that the effect of lipid attachment can be best understood by considering the temperature-dependent interactions between lipids and the ELP, with medium-length fatty acids providing the hydrophobic interactions needed to allow the phase transition temperature to be tuned. In contrast, short-chain fatty acids have too weak of an interaction with the ELP, and long-chain fatty acids are too hydrophobic and prefer to interact exclusively with each other, irrespective of the temperature.

## Experimental Section

### Materials

Carboxylic acids (C2:0-C16:0), triethylamine, apomyoglobin, aldolase, sinapinic acid, trifluoroacetic acid (TFA), and syringe filters were purchased from Sigma-Aldrich (St. Louis, MO). Acetonitrile and SnakeSkin dialysis tubing with a 3.5 kDa nominal molecular weight cutoff, tryptone, yeast extract, sodium chloride, kanamycin, and phosphate-buffered saline (PBS) were purchased from Thermo Fisher Scientific (Rockford, IL). Poly(*N*-isopropylacrylamide) (PNIPAM) and poly(*N*,*N*-dimethylacrylamide) (PDMA) were purchased from Polymer Source (Quebec, CA). Chemically competent BL21(DE3) cells were purchased from New England Biolabs (Ipswich, MA). Deionized water was obtained using a Milli-Q system (Millipore SAS, France). All chemicals were used as received without further purification.

#### Protein Expression

**Figure 2:**
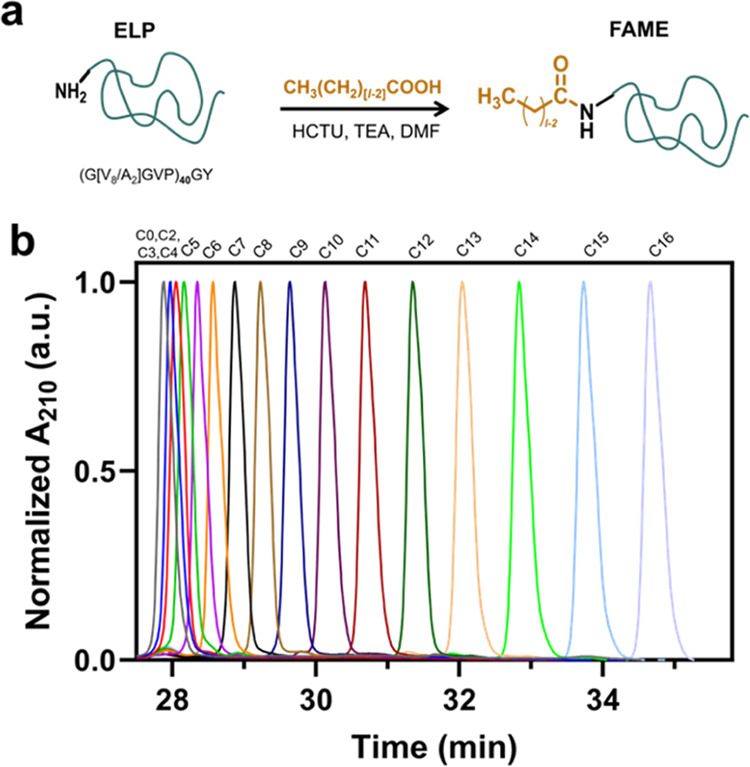
Construction and characterization of the FAME library. (a) Schematic of the chemical reaction used to modify ELPs with saturated fatty acids. (b) RP-HPLC traces of the FAME library indicate an increase in retention time as the lipid length is increased.

The unmodified protein substrate, ELP, was expressed in the *E. coli* BL21(DE3) strain. Bacterial cultures in sterile 2x YT medium supplemented with kanamycin (45 μg/mL) were cultivated in an incubator shaker (37 °C, 200 rpm) until their optical density (OD_600_) reached 1. The expression of ELP was induced by adding isopropyl β-d-1-thiogalactopyranoside (IPTG) to a final concentration of 1 mM. After 18 h, cells were harvested by centrifugation at 3745*g* and 4 °C for 30 min. The bacterial pellet was resuspended in phosphate-buffered saline (pH 7.4, 10 mL/L expression culture) and sonicated (75 W, 3 min) on ice. The unmodified ELP was purified by LLPS using the inverse transition cycling method.^42^ The isolated ELP was dialyzed against water overnight at 4 °C, and the dialysis retentate was subsequently lyophilized and stored at −20 °C. The N-terminal sequence (and the removal of methionine corresponding to the start codon) was verified by Edman degradation (Figure S1). The purity of unmodified ELP was confirmed by using RP-HPLC (Figure 2), and the identity of the protein was confirmed by MALDI-TOF (Figure S2).

### Conjugation of Lipids

To generate the constructs used in this study, we modified the N-terminal glycine residue of the model protein substrate by using fatty acids with different carbon chain lengths. To do so, the fatty acids (6 mmol) were dissolved in dimethylformamide (DMF, 1 mL) and subsequently activated in situ by incubating with a coupling reagent (6 mmol), O-(1H-6-chlorobenzotriazole-1-yl)-1,1,3,3-tetramethyluronium hexafluorophosphate (HCTU) and a base (12 mmol, triethylamine, TEA) for 20 min. Subsequently, ELP (3 mmol) was added to the reaction mixture, which was stirred for 3 h. The reaction was quenched by adding water (9 mL) and subsequently dialyzed to remove the unmodified starting material.

### RP-HPLC

Analytical RP-HPLC was performed on a Shimadzu LC-2030 instrument equipped with a UV–vis detector using a C18 column (Phenomenex Jupiter, 5 μm, C18, 300 Å, 250 mm × 4.6 mm). All samples were filtered through a 0.2 μm PVDF filter before analysis. The mobile phase was a mixture of water and acetonitrile containing 0.1% trifluoroacetic acid (TFA), and it was delivered at a flow rate of 1 mL/min. The acetonitrile content was linearly increased from 0 to 90% over 40 min.

### Matrix-Assisted Laser Desorption Ionization Time-of-Flight

MALDI-TOF-MS was conducted on a Bruker Microflex LRF instrument with a microScout ion source. A saturated solution of sinapinic acid (10 mg/mL in 70% acetonitrile in water +0.1% TFA) was used as the matrix. Samples were prepared by a serial dilution of the matrix. The solutions were dispensed onto a sample plate and dried at room temperature. Apomyoglobin (MW = 16,952.27 Da) and aldolase (MW = 39, 211 Da) were used to calibrate the instrument.

### Variable-Temperature Turbidimetry

To measure cloud points, FAMEs were dissolved in phosphate-buffered saline (PBS) at various concentrations (1–500 μM). Solutions were heated at a rate of 1 °C/min from 15 to 65 °C, and turbidity was measured by recording the scattering (absorbance at 350 nm) as a function of temperature. Above a critical temperature, the ELP becomes insoluble in water and turbidity starts to increase. Given the sharp transitions observed in this system, we determined the phase separation temperature as the inflection point of the turbidity plot and the maximum of the first derivative plot (Figure S3). The reversibility of the phase transition was confirmed by monitoring the absorbance of the sample while cooling the solution to 15 °C at a rate of 1 °C/min (Figure S4).

### Pyrene Fluorescence Assay

Critical micelle concentration (CMC) values were determined by using a pyrene-based fluorescence assay on a FluoroMax-4 spectrofluorometer (Horiba Jobin Yvon Inc.). A 12 mM pyrene stock solution in ethanol was diluted to 0.72 μM in PBS and sonicated for 15 min for homogenization. Serial dilutions of the protein samples, 0.001–200 μM, were prepared using the pyrene–PBS solution. The fluorescence emission of pyrene in these solutions was recorded in the range of 360–400 nm, with excitation set at 334 nm. After subtracting the emission intensity of a blank sample (containing only pyrene), the intensity ratio *I*_1_/*I*_3_ was calculated, where *I*_1_ and *I*_3_ are the intensities at 372 and 383 nm, respectively, and plotted against protein concentrations on a semilogarithmic scale. Data points were fitted with two linear equations; the intersection point was identified as the CMC value.

### Dynamic Light Scattering (DLS)

Experiments were conducted using a Zetasizer Ultra (Malvern Panalytical), equipped with a backscattering (173°) detector. Protein samples (100 μM in PBS) were filtered through a 0.22 μm poly(vinylidene fluoride) (PVDF) filter (Durapore). Each sample was measured in triplicate, with 11 acquisitions of 5 s each, at 20 °C. The DLS correlation functions were analyzed with the general purpose algorithm in ZS XPLORER software (version 1.2.0.91, Malvern Panalytical) to determine the intensity distribution of particle sizes.

### Size Exclusion Chromatography (SEC)

Size exclusion chromatography (SEC) was performed on a Shimadzu LC-2030 with a UV–vis detector using PBS as the mobile phase. Protein samples (50 μM) were analyzed by using Shodex OHpak SB-804 HQ and PROTEIN KW-804 columns.

### Temperature Gradient Microfluidics (TGM)

The kinetics of the ATPS formation were measured by using a temperature gradient apparatus. Briefly, a pair of heating and cooling elements (resulting in a linear temperature gradient between the elements) were fixed in a temperature-controlled chamber under a dark-field microscope. Samples (10 mg/mL in PBS) were loaded into rectangular borosilicate glass capillary tubes (VitroCom, Inc.) of dimensions 5 cm × 1 mm × 0.1 mm (length × width × height) and sealed with wax to prevent evaporation and convection. Capillary tubes were placed parallel to the direction of the thermal gradient. Light scattering from protein solutions was monitored using a digital camera (DS-Qi2, Nikon) under an optical microscope (SMZ18, Nikon) equipped with dark-field optics. Two reference solutions with different cloud point temperatures were employed alongside the samples to calibrate the temperature gradients. The reference solutions contained 10 mg/mL of poly(*N*-isopropylacrylamide) (PNIPAM) or 15 mg/mL of poly(*N*,*N*-dimethylacrylamide) (PDMA) with a given salt (NaCl or Na_2_SO_4_) concentration in water. The cloud point temperature was previously determined by using a melting point apparatus that measured the scattering intensity as a function of temperature.

Light-scattering data were transformed into normalized intensity versus time plots in each temperature region along the temperature gradient to analyze the kinetics of ATPS formation by fitting the data to a first-order or second-order rate equation (Figure S5). The light-scattering decay profiles were best fit to a single-exponential decay function (eq 1)
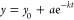
1where *y* is the intensity of the scattered light, *y*_0_ is the background intensity, *a* is a proportionality factor, and *k* is the rate constant for the process. The temperature dependency of ATPS rate constants was analyzed via the Arrhenius equation (eq 2)
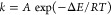
2where *k* is the rate constant, *A* is the prefactor, Δ*E* is the apparent activation energy, *R* is the gas constant, and *T* is the temperature in Kelvin. By plotting ln *k* versus 1/*T*, Δ*E* can be obtained from the slope of the linear regression curve.

### Data Analysis and Statistics

Statistical analyses, curve fitting, and 3D plotting were conducted using GraphPad Prism (version 9.2) or Origin Pro 2023 (version 10) software suites. TGM data were processed using a Python program previously developed in-house.^43^

## Results

### Synthesis and Characterization of FAME Library

A model ELP with a constant chemical composition was expressed in *Escherichia coli* and purified by utilizing its temperature-triggered phase separation behavior. This ELP contains 40 pentapeptides of GXGVP, in which X is a mixture of Val and Ala in a ratio of 80:20. FAMEs are produced by chemically conjugating fatty acids of different lengths (*l* = 2–16) to the N-terminal glycine residue in solution, as described in the Experimental Section (Figure 2a). The identity of all constructs was verified using MALDI-TOF-MS (Figure S2).

### Mean Hydrophobicity of Constructs Increases with Lipid Length

RP-HPLC was used to compare the hydrophobicities of the FAME constructs (Figure 2b). Using a C18 column and a linear gradient of nonpolar solvents, the elution time of each construct can be used as a proxy for hydrophobicity. In each case, we anticipated that column interactions occur primarily between the conjugated lipid and the hydrophobic alkyl chains attached to the resin. The retention time of each construct increased as the lipid chain length increased because, under HPLC conditions, the organic solvents and low porosity of the columns can dissociate FAMEs into unimers. In this study, we observed that the relative retention time of constructs fit very well to a quadratic equation (Figure S6), in agreement with previous reports for modeling the elution times of fatty acids and hydrophobic peptides.

### *T*_ph_ Can Be Quantitatively Correlated to the Lipid Length

**Figure 3:**
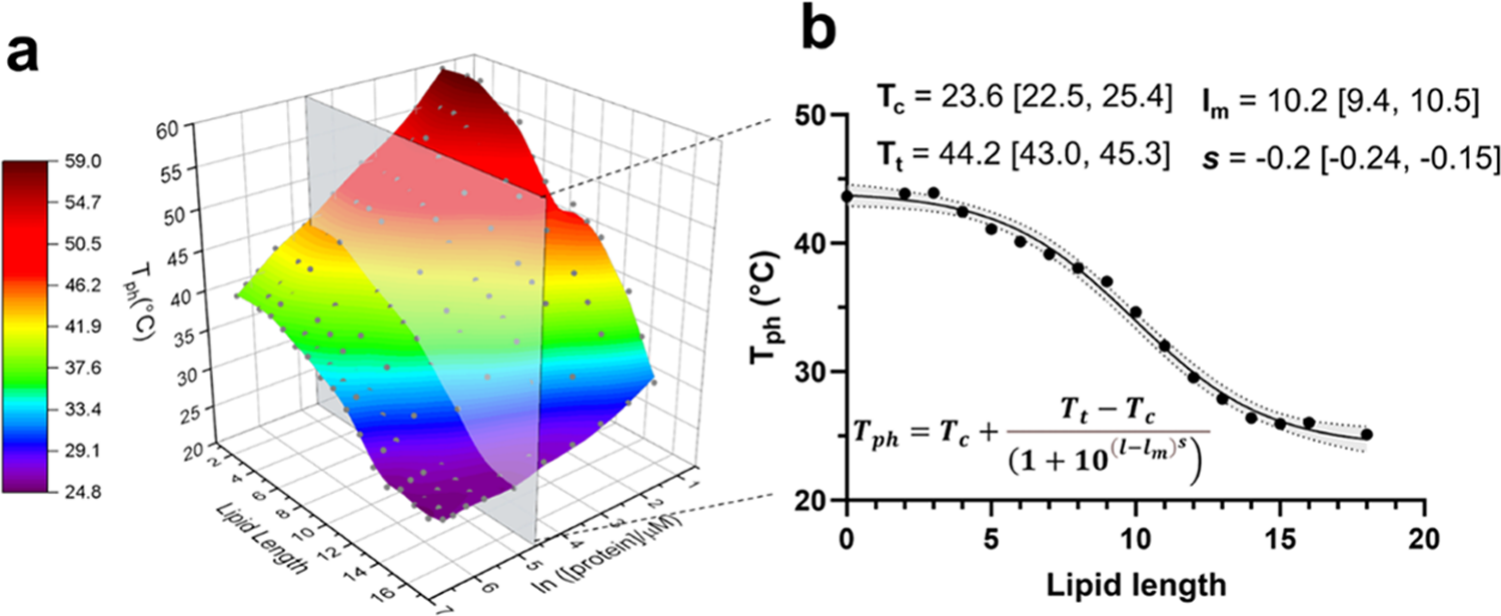
Temperature–composition–concentration phase diagram for FAMEs. Panel (a) shows a 3D surface plot of the cloud point temperature as a function of the lipid length and FAME concentration, constructed from a variable-temperature turbidimetry assay. (b) At a constant concentration (100 μM), *T*_ph_ exhibits a nonlinear dependence on the lipid length. The shaded region represents the 95% CI for the fitted 4PL model (variable slope, dose–response curve).

To quantify the effect of lipidation on the ELP phase behavior, we first constructed a partial temperature–composition phase diagram for the FAME library using variable-temperature turbidimetry experiments (Figure 3a). This was accomplished by monitoring the turbidity of FAME solutions prepared at different concentrations (1–500 μM in PBS buffer) as a function of solution temperature (Figure S7). FAMEs, like unmodified ELPs, exhibited LCST behavior as the solution turned turbid once the temperature was increased above the *T*_ph_. *T*_ph_ represents the temperature above which the balance of hydrophobic and hydrophilic interactions between the protein chain and water is shifted in favor of the collapsed state. Increasing the lipid length increases the unfavorable interactions of the protein chain with water and should therefore reduce the *T*_ph_ (Figure 3b). Intriguingly, this decrement was not linearly related to the lipid length despite the uniform increase in bulk hydrophobicity that each additional methylene group provides. As described below, these results suggest that the effect of lipid physicochemistry is not only limited to an increase in the mean hydrophobicity for each construct as suggested by RP-HPLC but also that the supramolecular assembly (micellization) of FAMEs may be altered.

The changes in *T*_ph_ as a function of lipid length exhibited a sigmoidal relationship between two limiting regimes: (1) the upper bound for *T*_ph_ was the transition temperature (*T*_t_) of the unmodified ELP (at a similar concentration) and (2) the lower bound of *T*_ph_ was the transition temperature of the unmodified ELP with a similar guest residue composition at the limit of high molecular weight (*T*_c_). Therefore, we fitted these data to a four-parameter logistic curve (4PL), (eq 3)
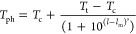
3

In this equation, *l*_m_ denotes the sigmoid’s midpoint, while the Hill slope, *s*, describes the steepness of the curve, indicating the degree of cooperativity between the increasing number of methylene groups and their impact on *T*_ph_. A representative fit (and 95% confidence interval) for [FAME] = 100 μM is shown in Figure 3b, and the results for other concentrations are reported in the Supporting Information (Table S2 and Figure S8). For a broad range of concentrations (50–500 μM), the values for fitted *l*_m_ and *s* are approximately 9–10 and −0.2. The observed negative cooperativity indicates that increasing the lipid length promotes the micellization of FAMEs (Figure S8), which reduces unfavorable interactions between the lipid and water at the expense of altering the entropy of phase separation for micelle association to form coacervates. This aligns with critical micelle concentration (CMC) studies showing a decreasing CMC trend from 40 to 3 μM as lipid length increases from 3 to 16 (Figures S9 and S10) and is corroborated by DLS (Figure S11).

### Monitoring the Kinetics of ATPS Formation Using Temperature Gradient Microfluidics (TGM)

**Figure 4:**
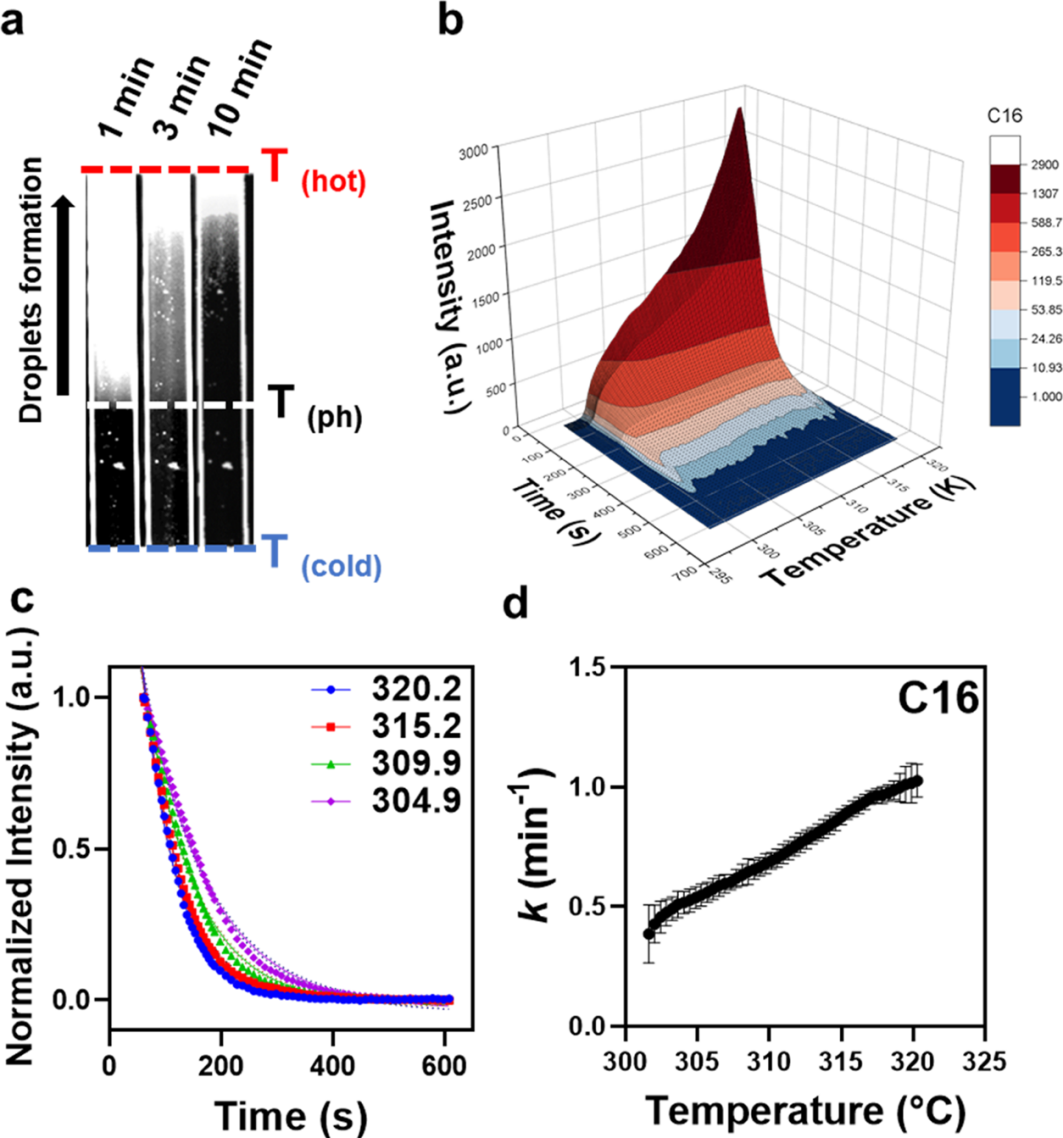
Representative kinetic analysis of ATPS formation for C16 FAME. (a) Dark-field images of a single microfluidic channel on a linear temperature gradient at *t* = 1, 3, and 10 min. (b) 3D plot of scattering intensity as a function of time and temperature. (c) Representative curve fits for intensity decay at four temperatures. All four curves are fit to a single-exponential decay. (d) The rate constant, *k*, obtained from fitting the intensity-decay data to first-order kinetics as a function of temperature. The error bars are standard deviations of 6 measurements. See Figures S13–S27 for an analysis of unmodified ELP and C2–C15 FAMEs. Concentration is 10 mg/mL.

After investigating the correlation between the lipid length and thermodynamic phase boundaries (indicated by the lower critical solution temperature) of the ELP, we investigated the influence of lipid length on the kinetics of ATPS formation. In each case, a solution of FAME in PBS at 10 mg/mL (∼600 μM ≫ CMC_*l*≥3_) was introduced into the microfluidic channel below *T*_ph_. The device was then placed on a linear temperature gradient spanning 288–321 K, and ATPS formation was monitored using dark-field microscopy (Figure 4a). The jump in temperature caused the ELP to phase-separate almost immediately on the warmer side of the device. At *T* < *T*_ph_, the protein is soluble in water with very little light scattering (dark regions of the channel). However, at *T* > *T*_ph_, the protein demixes from the aqueous solution, resulting in the formation of a cloudy suspension of protein droplets. These droplets were sufficiently large to scatter light (the white region of the channel). Over time, the turbid region started to shrink, indicating that the droplet suspension completed ATPS to form two distinct phases. As shown in Figure 4b, TGM enabled us to simultaneously measure the changes in turbidity over time across a range of temperatures and to obtain kinetic data for ATPS formation in the FAME library. The ability to monitor the decrease in turbidity as a function of both time and temperature allowed us to observe the temperature dependence of the ATPS kinetics (Figure 4c). These data provide valuable insights into the mechanistic pathways involved in the phase separation process and reveal how changes in lipid length alter the activation energy of the individual mechanistic steps.

### ATPS Formation of FAMEs Is Governed by Coalescence, Regardless of Lipid Chain Length

A colloidal suspension of protein droplets can undergo ATPS via two primary mechanisms: coalescence and Ostwald ripening (Figure 1).^44,45^ Coalescence refers to the fusion of two individual droplets, resulting in the formation of larger droplets that ultimately settle to the bottom of a microfluidic channel to form a protein-rich phase. By contrast, Ostwald ripening involves the transfer of individual ELP molecules from smaller droplets to larger ones by way of the surrounding bulk solvent. This phenomenon occurs because of the differences in solubility and surface energy with curvature, leading to the growth of larger droplets at the expense of smaller ones. Ultimately, Ostwald ripening affects the size distribution of droplets within the system over time.

At sufficiently high concentrations of droplets, these mechanisms have different kinetic signatures, as coalescence exhibits first-order kinetics, while Ostwald ripening exhibits second-order kinetics.^46^Figure 4c shows line scans from a representative sample with a 16-carbon long lipid chain (C16) plotted against both temperature and time. To confirm which mechanism was operating, we fitted the decay in the intensities to both first- and second-order models. For all FAMEs, data fit first-order kinetics (one-phase exponential decay) well (*R*^2^ > 0.98) and did not fit the second-order kinetics. The specific equation for the intensity decay was *I*(*t*) = *I*_0_ exp(*−kt*).

Based on these results, ATPS formation of FAMEs primarily occurs through the coalescence of smaller droplets into larger ones, resulting in the creation of two thermodynamically stable phases. This finding is consistent with previous research on ELPs, which also showed that coalescence is the primary mechanism for ATPS in the absence of surfactants.^38^

The simultaneous measurement of the ATPS rate over a temperature gradient enabled us to determine *k* as a function of temperature. For example, the rate of ATPS production increased with an increasing temperature for C16 (Figure 4d). Because each construct had a different value for *T*_ph_, we compared the temperature dependencies of the ATPS rate instead of the absolute rate constants. This type of analysis provides a mechanistic window into ATPS formation and showed that the lipid length influences the operating mechanism of ATPS.

### Lipid Length Alters Temperature Dependence of ATPS Kinetics

**Figure 5:**
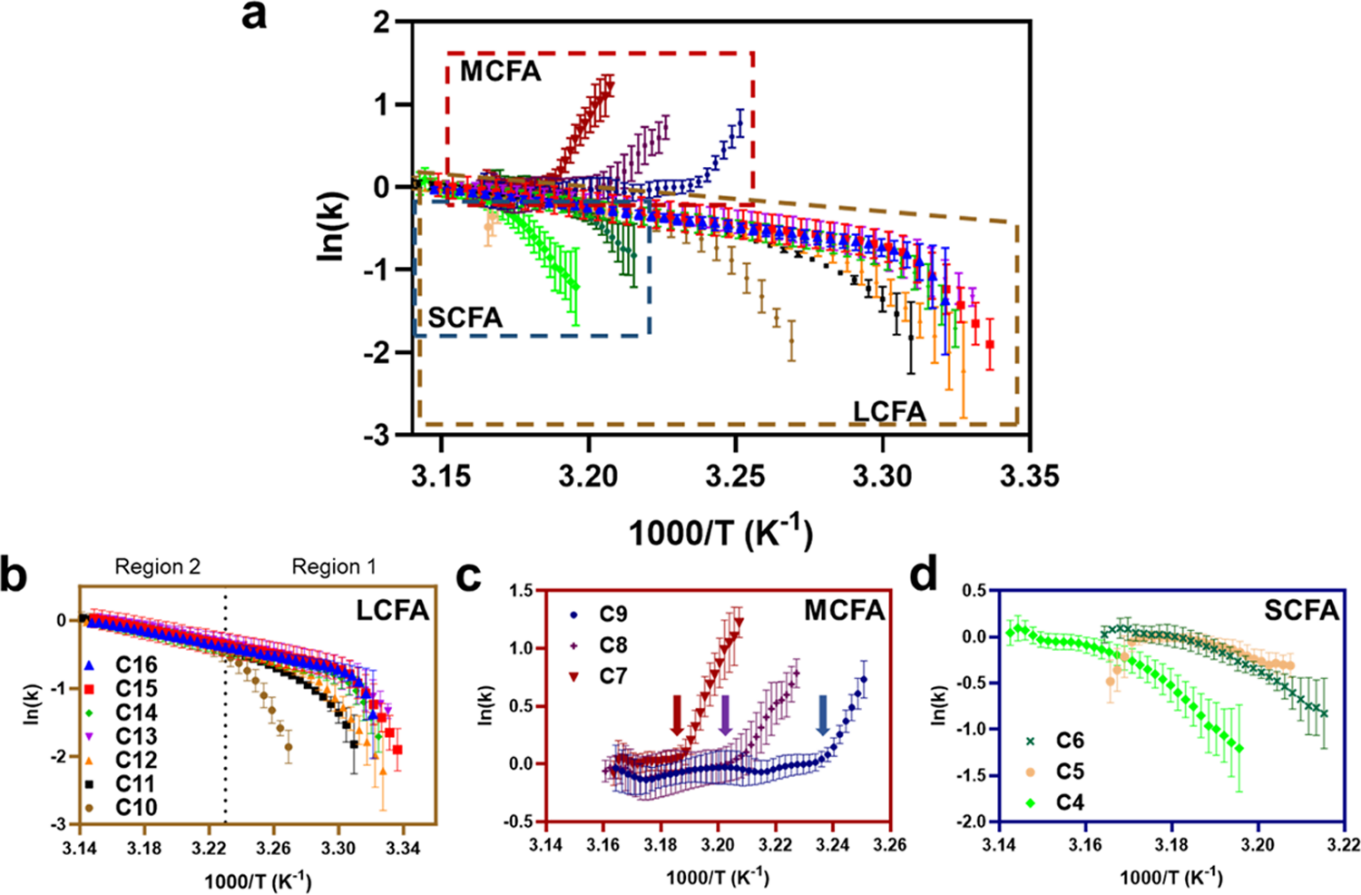
(a) Arrhenius plot analysis of FAMES ATPS exhibited three distinct regions based on the length of the lipid tail. (b) Long-chain fatty acids. The dotted line is placed as a visual guide to separate the two regions with distinct temperature dependencies for C10. (c) Medium-chain fatty acids. The arrows are placed to denote the onset of the observed non-Arrhenius behavior. (d) Short-chain fatty acids. The error bars are standard deviations of 4–6 measurements. See Table S3 for the apparent activation energies for each construct.

To investigate the temperature dependence of the ATPS rate constant, we used the Arrhenius model (eq 2)
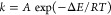
Here, Δ*E* is the apparent activation energy and *T* is temperature in degrees Kelvin. *R* represents the gas constant, and *A* is a prefactor. Figure 5a shows the results of Arrhenius analysis for the FAME library (ln* k*_ATPS_ is plotted against 1000/*T*) and highlights the effect of lipid physicochemistry on the temperature dependencies of the ATPS rates. As can be seen, these plots can be divided into three distinct regions based on the length of the fatty acid chain: long-chain fatty acids (LCFA, C10–C16), medium-chain fatty acids (MCFA, C7–C9), and short-chain fatty acids (SCFA, C4–C6). Expansions of the three distinct regions are provided in Figure 5b–d.

### Long-Chain Fatty Acids (10 ≤ *l* ≤ 16)

Two distinct temperature-dependent regions are observed in the data (Figure 5b). Region 1 occurs at temperatures slightly above the cloud point and is characterized by a slow ATPS rate (region to the right of the vertical dotted line). In this region, the rate of ATPS formation accelerated in a nonlinear fashion as the temperature increased. In region 2 (to the left of the vertical dotted line), we observed a clear “Arrhenius-like” dependence of the rate of ATPS over a broad temperature range. Fitting the data in this region to a linear regression model yielded an activation energy of 10.2 kcal/mol (Table S3), which is several-fold smaller than the apparent activation energy for the first region (∼40 to 60 kcal/mol). The lower value is in agreement with the activation energy observed for the ATPS formation of α-elastin (10.4 kcal/mol)^46^ and other ELPs (10.0–10.8 kcal/mol) that undergo a phase transition via the coalescence mechanism.^38^ The higher activation energy values at lower temperatures (region 1) may reflect the slow process of droplet generation for lipidated samples near the phase boundary at colder temperatures. As the temperature is increased, however, more ELP droplets are formed, which can undergo ATPS.

### Medium-Chain Fatty Acids (7 ≤ *l* ≤ 9)

Here, we again observed two distinct kinetic regimes, but their temperature dependencies were strikingly different from the behavior of the long-chain fatty acids (Figure 5c): in one region corresponding to elevated temperature (*T* ≫ *T*_ph_), the overall rate of the ATPS did not change significantly with temperature. More strikingly, the temperature dependences near *T*_ph_ exhibited “anti-Arrhenius” behavior; that is, the rate of ATPS increased as the temperature was reduced. This results in an apparent activation energy of −70 to −131 kcal/mol (Table S3). This crossover behavior shifted to colder temperatures as the lipid length was increased (denoted with arrows in Figure 5c), which suggests that the physicochemical properties of lipids influence the mechanism of ATPS, likely by altering the balance of temperature-dependent attractive interactions between the lipid and ELP.

### Short-Chain Fatty Acids (4 ≤ *l* ≤ 6)

The temperature dependences of short-chain fatty acids were similar to those observed with long-chain fatty acids (Figure 5d). The rate of ATPS was slow near *T*_ph_ but increased with temperature. Despite the increased curvature (nonlinearity) in the Arrhenius plot, the apparent activation energy was 12.0–13.7 kcal/mol, which is consistent with the value for the coalescence of ELPs.

Finally, an unmodified ELP and FAMEs modified with the shortest lipids (C2 and C3) did not undergo significant ATPS (Figures S13–S15) under conditions that are comparable to Figure 3b. In these cases, LLPS occurred, but ATPS formation did not come to completion. These results demonstrate that in this system a minimum lipid length of four carbons is needed before the lipid can alter the phase separation process of ELPs.

## Discussion

### Correlation between Lipid Physicochemistry and ELP Phase Boundaries

Our findings indicate that lipids with *l* ≥ 4 can reduce the ELP phase boundary, and this reduction correlates sigmoidally with lipid length. We propose the following mechanism to explain the effects of conjugated lipids on ELP phase boundaries. Lipidation changes the Gibbs free energy of liquid–liquid phase separation by increasing unfavorable interactions of FAMEs with water. As the lipid length is increased above a critical threshold, the attractive homotopic interactions between the lipids favor the micellization of FAME chains. This will reduce unfavorable lipid–water interactions but it also reduces the entropy needed for ATPS formation. *T*_ph_ represents a temperature at which these interactions and the ideal entropy of mixing are in balance. As the lipid length increases, *T*_ph_ decreases sigmoidally until it reaches a critical temperature limit corresponding to an ELP of high molecular weight and concentration.

### Correlation between Lipid Physicochemistry and Aqueous Two-Phase System (ATPS) Kinetics

To advance the utilization of water-in-water (w/w) emulsions with customized stability, understanding the mechanisms governing emulsion formation and breakdown is essential. By understanding these mechanisms, we can manipulate the molecular properties of proteins to control emulsion formation and stability.

The TGM technique enables the rapid and simultaneous measurement of ATPS kinetics under varying conditions. It has been applied to the study of protein phase separation, such as α-elastin^46^ and ELPs,^38^ and has revealed that ATPS formation in these intrinsically disordered proteins predominantly occurs through a coalescence mechanism. Interestingly, the presence of surfactants can alter this dynamic, reducing the coalescence rates and potentially shifting the dominant ATPS mechanism toward Ostwald ripening.

Our results demonstrate that the kinetics of ATPS formation for the FAMEs studied here fit a first-order model, suggesting that the dominant mechanism for their ATPS formation in this case is also coalescence. As the lipid length increased, the observed decay profiles better fit single-exponential decay, indicating that increasing the lipid chain length disfavors the Ostwald ripening pathway. This effect contrasts with the influence of small-molecule surfactants on the ATPS of macromolecules, where surfactants—by lowering the surface energy of droplets—hinder coalescence and thereby encourage Ostwald ripening. However, this dynamic changes when lipid chains are directly conjugated to ELPs. This conjugation leads to the formation of micelles, significantly enhancing the coalescence process. In such cases, an ELP linked to a lipid chain is less likely to detach from a micelle, thus favoring coalescence over Ostwald ripening. Furthermore, the large size of these micelles impedes their transfer to the solution, elevating the activation energy required for Ostwald ripening, which scales with the micelle’s surface area.^47−49^ In essence, lipidation restricts the transfer of protein monomers from small droplets into the solution, effectively increasing the activation energy needed for Ostwald ripening as the lipid length increases. This hypothesis aligns with the observed correlation between the lipid physicochemical properties and critical micelle concentration.

Arrhenius plots were used to analyze the temperature dependence of the ATPS rate constant. Arrhenius plots offer valuable insights into the molecular mechanism of a process by revealing how temperature influences reaction rates and providing information about activation energies and reaction pathways. Lipid physicochemistry clearly alters the temperature dependence of ATPS rates with three distinct behaviors observed based on the length of the lipid (Figure 5). Specifically, sufficiently long-chain fatty acids exhibited similar behaviors (Figure 5b). In all cases, the ATPS rate was initially slow at temperatures slightly above *T*_ph_. The ATPS rate increased with the temperature and exhibited a traditional Arrhenius dependence over a broad range of temperatures. We determined the activation energy for the two-phase constructs to be ∼10.2 kcal/mol, which is in good agreement with our and other previous results obtained for unmodified elastins.^38^ This observation was consistent with the formation of stable micelles for these constructs. These micelles have similar ELP sequences at their corona, which may explain why a similar activation energy is obtained at the cloud point past a critical lipid length.

In contrast to the results for longer-chain lengths, FAMEs modified with medium-chain fatty acids exhibited an accelerated ATPS rate at colder temperatures—a non-Arrhenius type of behavior that shifted to lower temperatures as the lipid length was increased from C7 to C9. Remarkably, the addition or subtraction of one methylene group was sufficient to significantly modulate this behavior. We propose that this observation is due to temperature-dependent hydrophobic interactions between lipids and ELP, as these hydrophobic interactions depend nonlinearly on temperature.

For medium-chain fatty acids, the balance between homotopic and heterotopic interactions is delicate and can be altered as a function of temperature because the ELPs are dehydrated at elevated temperatures.^50^ Long-chain fatty acids are too hydrophobic, and in the experimental range that was investigated, they prefer to interact exclusively with each other, while the interaction between the short-chain fatty acids and ELPs is too weak, regardless of temperature. This adjustable interaction mirrors patterns seen in computational models^41^ and other biological systems, such as myristoyl-switches,^51^ that internalize lipid post-translational modifications in their hydrophobic domains.

We note that the temperature dependence of ATPS is influenced by a combination of macroscopic (droplet coalescence) and microscopic events (e.g., the dynamic equilibria among unimers, oligomers, and micelles). Changes in the temperature can have a profound impact on these microscopic events. For instance, when the temperature surpasses the cloud point of the FAMEs, it can lead to a decrease in the protein concentration within the dilute phase, potentially falling below the critical micelle concentration, which itself is likely temperature-dependent.^52^ However, despite these intricacies, our major discovery highlights the remarkable sensitivity of ATPS to even subtle alterations in the physicochemical properties of lipids. This observation underscores a significant contrast between lipid-mediated oligomerization and protein-mediated oligomerization.^53,54^ The latter is typically characterized by sequence-specific interactions that precisely define stoichiometry and multivalency in phase-separating proteins.^55,56^ Considering these findings, we propose that lipid-mediated oligomerization may offer a more adaptable and dynamic mechanism for the on-demand regulation of phase separation.

## Conclusions

The primary goal of our study was to establish a foundational understanding of how lipidation affects the phase behavior of ELPs, a model of intrinsically disordered proteins with LCST behavior. Through our analysis of phase boundaries, we sought to shed light on the influence of saturated fatty acid chains on the thermodynamics and kinetics of LLPS. Our findings revealed that FAMEs undergo ATPS formation via a coalescence mechanism. Because coalescence requires the rupture of the interface between droplets, this discovery suggests that the molecular engineering of FAMEs to alter the viscoelasticity of droplets may offer a potential strategy to stabilize these coacervates without relying on surfactants.^57,58^

In future work, we intend to extend this approach to investigate how the composition of the ELP (length and guest residue hydropathy) and the sequence of lipidation sites affect the properties of FAMEs. Building on insights gleaned from temperature-dependent interactions between medium-chain fatty acids and ELP, we anticipate that these molecular parameters can be used to modulate the adhesive and cohesive interactions among lipids, proteins, and water, thus providing a molecular basis for regulating the properties of FAME coacervates. Concurrently, we will expand the scope of our investigation to encompass other disordered proteins, including those with an upper critical solution temperature such as resilin-like polypeptides.

Importantly, these thermodynamic principles may offer broader applicability, extending to proteins modified with different lipid types such as unsaturated fatty acids and sterols, which similarly induce supramolecular protein oligomerization.^59,60^ The ability to predict phase boundaries based on molecular structures holds promise for accelerating the design and application of this class of hybrid biopolymers. Emulating nature’s precise control over condensate formation and material properties provides a compelling alternative for manipulating the size and morphology of aqueous systems without relying on separate molecular surfactants. This scientific and technical advancement should open previously untapped opportunities for the development of all-aqueous emulsions characterized by structural hierarchies and dynamics that rival those observed in biological systems. These innovations hold great promise in the fields of nanotechnology and materials science.

## Supporting Information Available

Complete list of materials and suppliers, supporting tables, and supporting figures (PDF)
