# Supplementary material for: Modulating Phase Behavior in Fatty Acid-Modified Elastin-like Polypeptides (FAMEs): Insights into the Impact of Lipid Length on Thermodynamics and Kinetics of Phase Separation
Source: J Am Chem Soc. 2024 Feb 14;146(8):5383–92. doi: 10.1021/jacs.3c12791 (PMC10910508; doi:10.1021/jacs.3c12791)
Supplement: Supplementary file 1 — ja3c12791_si_001.pdf [file ja3c12791_si_001.pdf]

Supplementary Information

Modulating Phase Behavior in Fatty Acid-Modified Elastin-Like Polypeptides (FAMEs): Insights into the Impact of Lipid Length on Thermodynamics and Kinetics of Phase Separation

Zhe Zhang, Christopher J. Lynch, Ying Huo, Somya Chakraborty, Paul S. Cremer, and Davoud Mozhdehi

psc11@psu.edu; dmozhdeh@syr.edu;

Table of Contents

1. Protein sequence S2
2. Supplementary tables S2
3. Supplementary figures S5
4. References S32

1. Protein sequence

ELP(V8/A2,40)

*(M)*GVGVPGVGVPGAGVPGVGVPGVGVPGVGVPGVGVPGAGVPGVGVPGVGVPGVGVPGV
GVPGAGVPGVGVPGVGVPGVGVPGVGVPGAGVPGVGVPGVGVPGVGVPGVGVPGAGVPG
VGVPGVGVPGVGVPGVGVPGAGVPGVGVPGVGVPGVGVPGVGVPGAGVPGVGVPGVGVP
GVGVPGVGVPGAGVPGVGVPGVGVPGY

2. Supplementary tables

Table S1. Theoretical molecular weight and observed m/z of each construct.

Construct	Theoretical M_w (Da)	Observed m/z $[M+H]^+$
ELP	16393.24	16396.89
C2-ELP	16435.29	16439.11
C3-ELP	16449.32	16446.64
C4-ELP	16463.35	16469.68
C5-ELP	16477.37	16477.07
C6-ELP	16491.4	16492.52
C7-ELP	16505.42	16509.16
C8-ELP	16519.45	16517.69
C9-ELP	16533.48	16527.01
C10-ELP	16547.5	16548.97
C11-ELP	16561.53	16563.04
C12-ELP	16575.56	16578.35
C13-ELP	16589.59	16594.48
C14-ELP	16603.61	16607.82
C15-ELP	16617.64	16621.17
C16-ELP	16631.67	16634.98

Table S2. Curve fitting parameters and goodness-of-fit statistics for a four-parameter logistic curve (4PL) correlating T_{ph} to lipid length calculated for FAMEs at various concentrations. These values are also plotted in Figure S8 to faciliate comparison.

Concentration (μM)	Fit Parameter (standard error)				R^2
500.0	T_c	24.7 (0.5)	l_m	8.8 (0.3)	0.984
	T_t	37.4 (0.5)	S	-0.3 (0.04)	
400.0	T_c	24.9 (0.5)	l_m	8.9 (0.3)	0.987
	T_t	38.5 (0.5)	s	-0.2 (0.04)	
300.0	T_c	25.0 (0.5)	l_m	9.0 (0.2)	0.991
	T_t	39.6 (0.4)	S	-0.2 (0.03)	
200.0	T_c	24.1 (0.8)	l_m	9.1 (0.3)	0.990
	T_t	42.6 (0.7)	S	-0.2 (0.02)	
100.0	T_c	23.6 (0.8)	l_m	10.2 (0.3)	0.993
	T_t	44.2 (0.6)	S	-0.2 (0.02)	
50.0	T_c	23.5 (1.4)	l_m	10.5 (0.4)	0.989
	T_t	47.7 (0.8)	S	-0.2 (0.02)	
25.0	T_c	22.5 (1.9)	l_m	11.4 (0.5)	0.989
	T_t	50.2 (0.9)	S	-0.2 (0.02)	
12.5	T_c	18.9 (5.1)	l_m	12.6 (1.0)	0.981
	T_t	53.8 (1.5)	S	-0.1 (0.03)	
6.3	T_t	11.3 (9.7)	l_m	14.7 (1.7)	0.989
	T_t	57.4 (1.6)	S	-0.1 (0.02)	
*3.2**	T_c	*-157.0 (1308.5)*	l_m	*32.7 (84.0)*	*0.970*
	T_t	*67.6 (13.4)*	*S*	*0.04 (0.1)*	
*1.6**	T_c	*-19.6 (38.7)*	l_m	*19.4 (4.0)*	*0.993*
	T_t	*63.3 (1.5)*	*s*	*-0.1 (0.02)*	

* The large standard error for the fitted parameters indicates that this empirical model is not a good descriptor of changes in T_{ph} as a function of lipid length for FAMEs at concentrations below the critical micellization concentration (Figure S10).

Table S3. Apparent activation was derived from the analysis of the ATPS rates using the Arrhenius model.

Construct	T_{low} – T_{high}	E_a	T_{low} – T_{high}	E_a
	K	Avg ± std. dev. (n= 3–6) kcal/mol	K	Avg ± std. dev. (n= 3–6) kcal/mol
C4	313 – 316	57.5 ± 1.3	316 – 321	13.3 ± 0.9
C5	312 – 314	32.7 ± 0.6	314 – 321	13.7 ± 0.8
C6	311 – 314	64.9 ± 0.8	314 – 321	12.0 ± 0.6
C7	312 – 314	-131.1 ± 1.3	314 – 321	-3.6 ± 0.3
C8	310 – 312	-73.5 ± 1.4	312 – 321	-7.7 ± 0.2
C9	308 – 309	-96.1 ± 2.9	309 – 321	-3.2 ± 0.3
C10	305 – 309	81.5 ± 1.8	309 – 321	10.5 ± 1.4
C11	302 – 306	59.9 ± 3.2	306 – 321	11.7 ± 1.1
C12	300 – 306	52.9 ± 1.0	306 – 321	10.2 ± 0.9
C13	300 – 302	37.8 ± 0.6	302 – 321	9.9 ± 1.5
C14	300 – 302	53.7 ± 2.9	302 – 321	10.1 ± 0.7
C15	299 – 302	54.6 ± 2.0	302 – 321	9.4 ± 0.4
C16	301 – 302	37.8 ± 3.7	302 – 321	9.0 ± 0.7

3. Supplementary figures

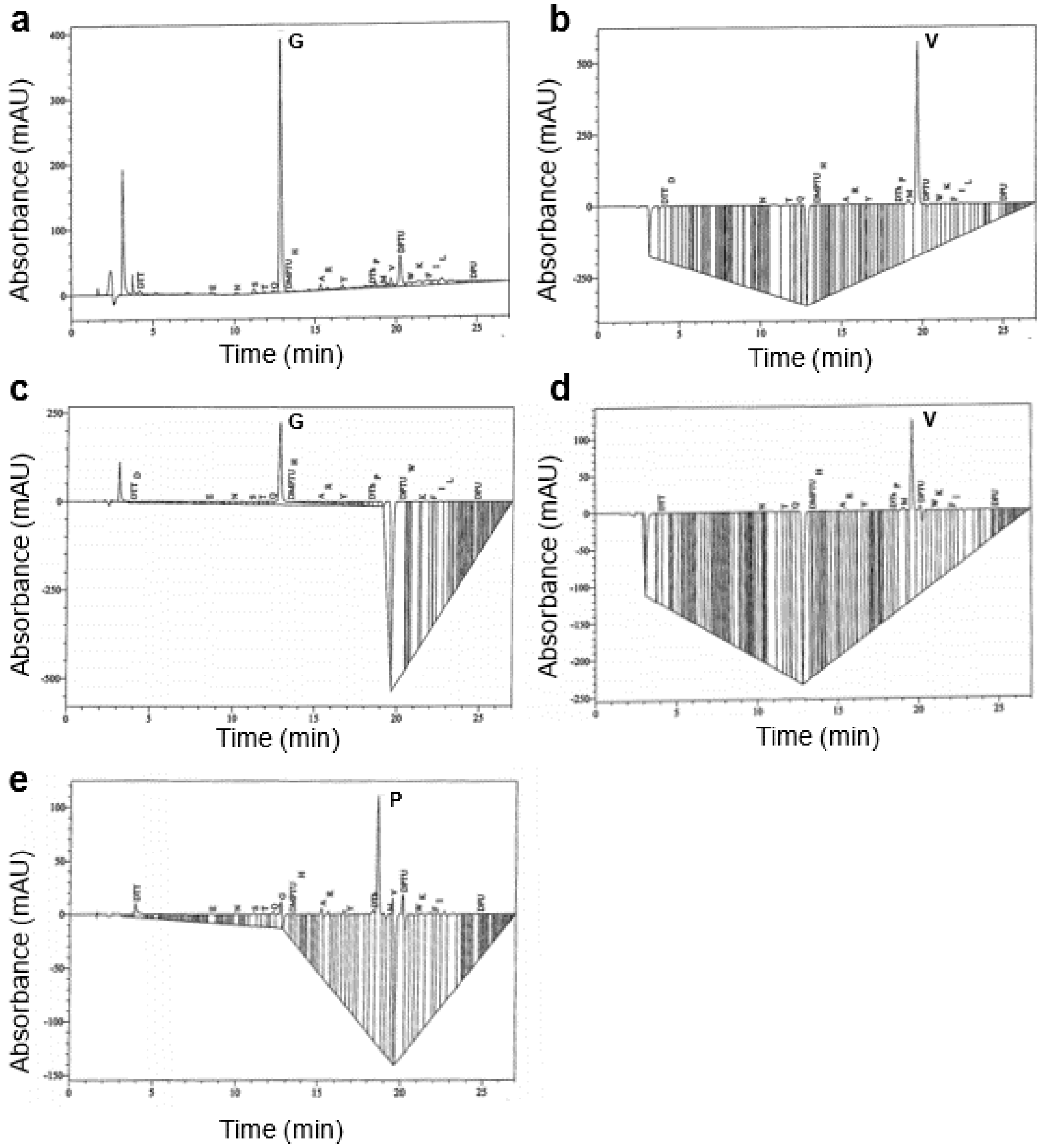

Figure S1. The chromatogram obtained for the first five cycles of Edman sequencing of recombinantly expressed ELP (a-e). Edman sequencing confirmed that the N-terminal sequence was GVGVP, consistent with the removal of the initial methionine residue.

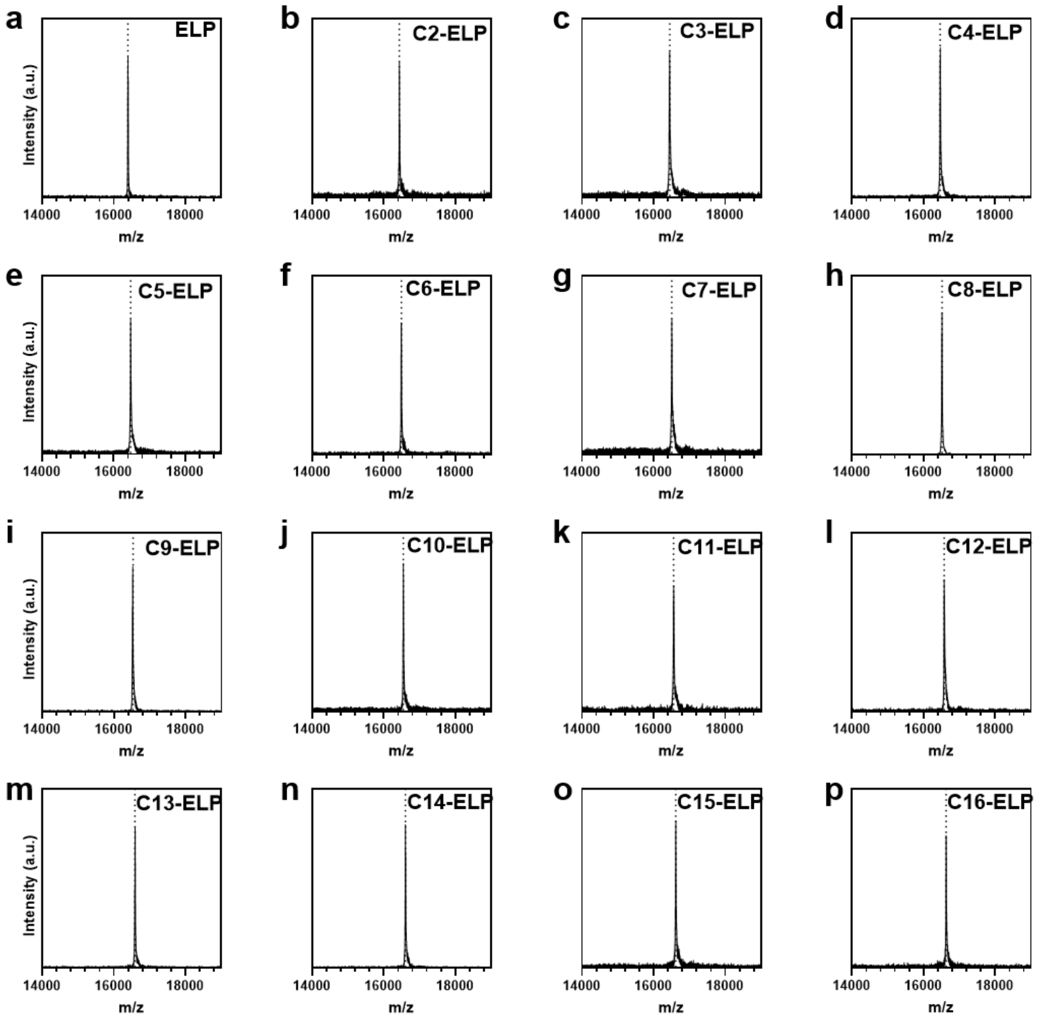

Figure S2. MALDI-TOF-MS spectra of the proteins used in this study. Vertical dotted lines denote each construct's theoretical molecular weight ($[M + H]^+$).

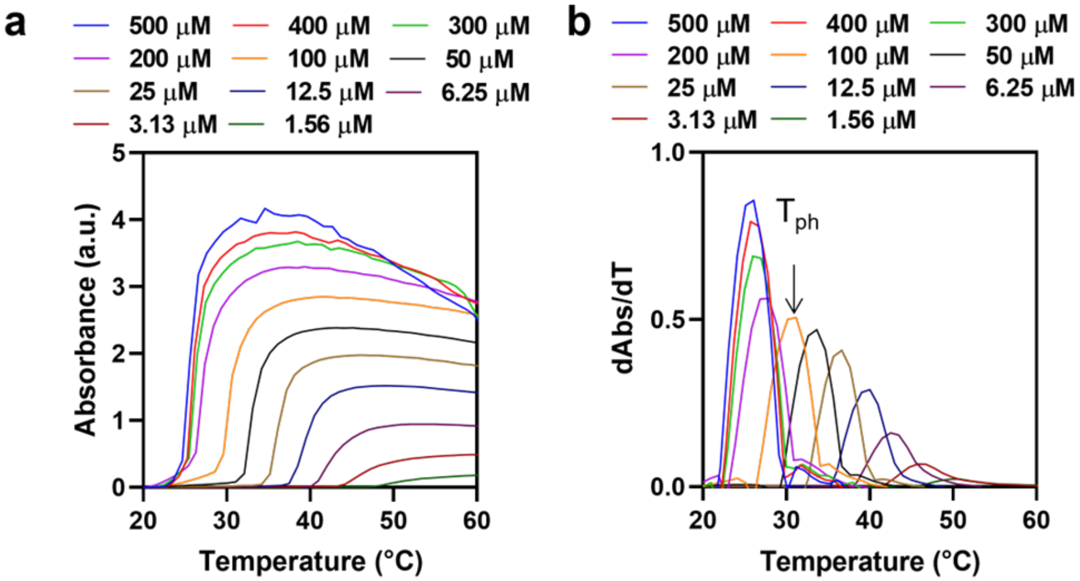

Figure S3. Representative turbidimetry plots used to determine phase-separation temperatures (T_{ph}) in Figure 3. (a) The turbidity of C12 FAME at different concentrations is plotted as a function of temperature. The absorbance sharply increased as the temperature was increased above T_{ph}. **(b)** The T_{ph} at each concentration was determined by identifying the peak in the first derivative plots. An arrow denotes the representative T_{ph} for C12 FAME at 100 μM.

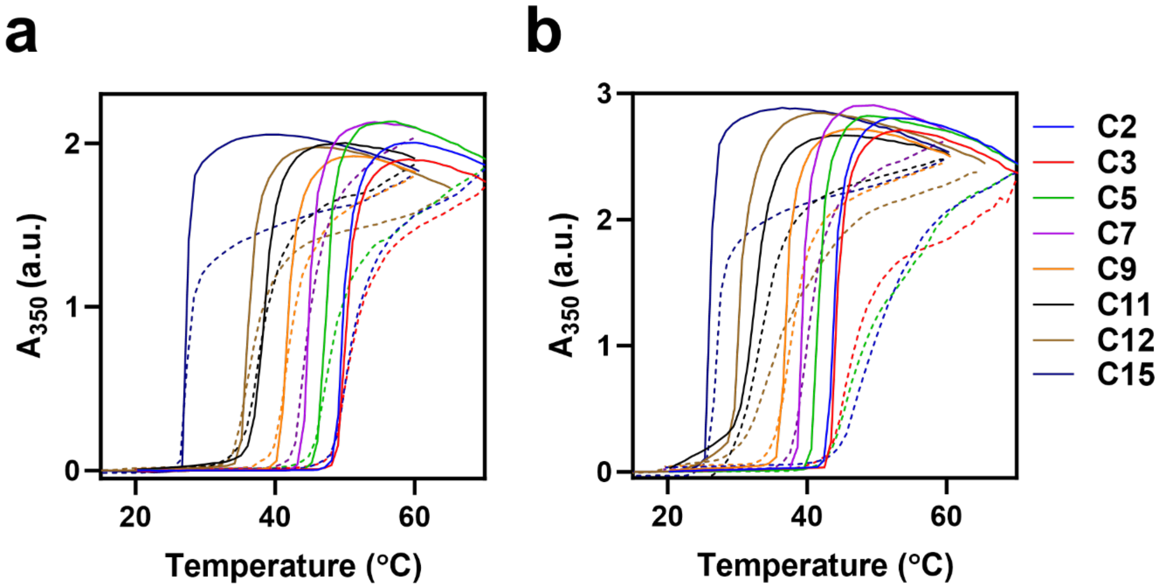

Figure S4. Reversible phase transition of representative FAMEs. The variable-temperature turbidimetry was conducted at (a) 25 µM; and (b) 100 µM. The phase-separation was found to be completely reversible, regardless of the lipid length.

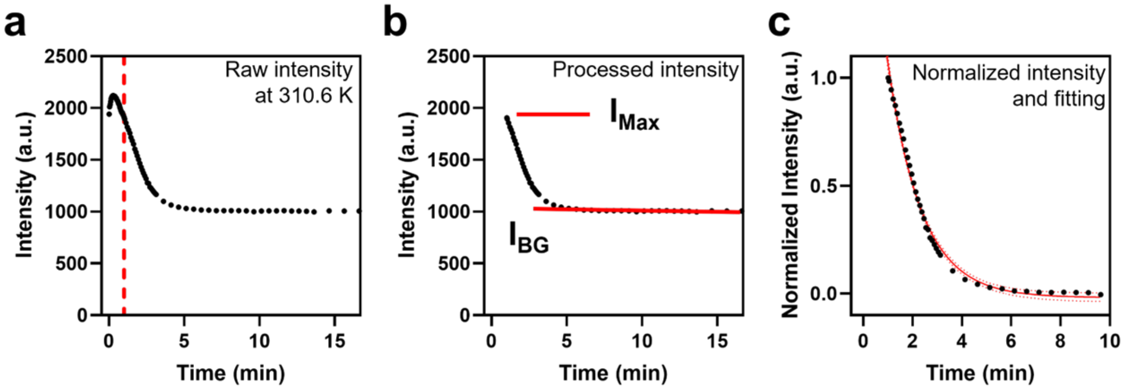

Figure S5. Representative workflow for processing thermal gradient microfluidics (TGM) data of C9 at 310.6 K. (a) The raw Intensity was obtained from the light-scattering data as a function of time at each temperature region. The data for the first 60 s (before the red dashed line), corresponding to the equilibration time of the temperature gradient, were discarded. **(b)** Data were baseline-corrected by subtracting the linear baseline corresponding to the average intensity of the pixels at 600–1000 s, corresponding to the time required for completion of the ATPS. **(c)** The data was normalized using the maximum intensity and background intensity. We plotted the light-scattering intensity for each temperature range within a 1-10 minute timeframe. Data after 10 minutes were discarded as there was little change in intensity after that point. After normalizing the data, it was fitted into either a first-order or second-order model. The figure illustrates a representative fit to the first-order model using a single-exponential decay, as shown by the red solid line. The dashed line represents the 95% confidence interval.

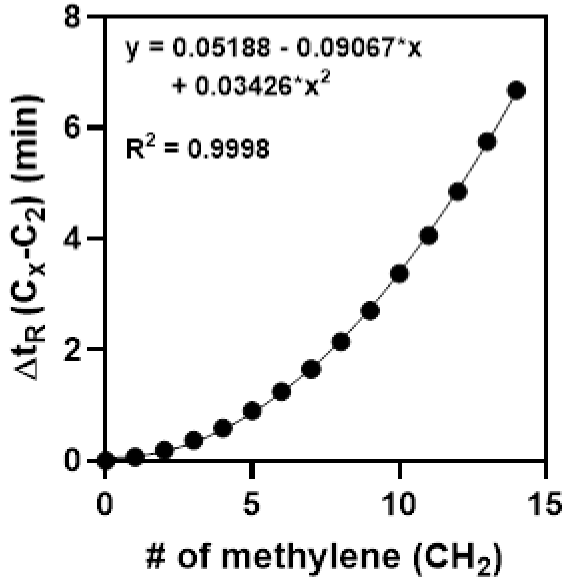

Figure S6. The retention time of FAMEs vs. the number of methylene groups can be well-described as a quadratic equation. The x-axis represents the number of methylene groups in the lipid, ranging from zero in C2 to 14 in C16. Such a relationship has been previously observed in the literature for modeling the elution time of fatty acids and hydrophobic peptides.[1, 2]

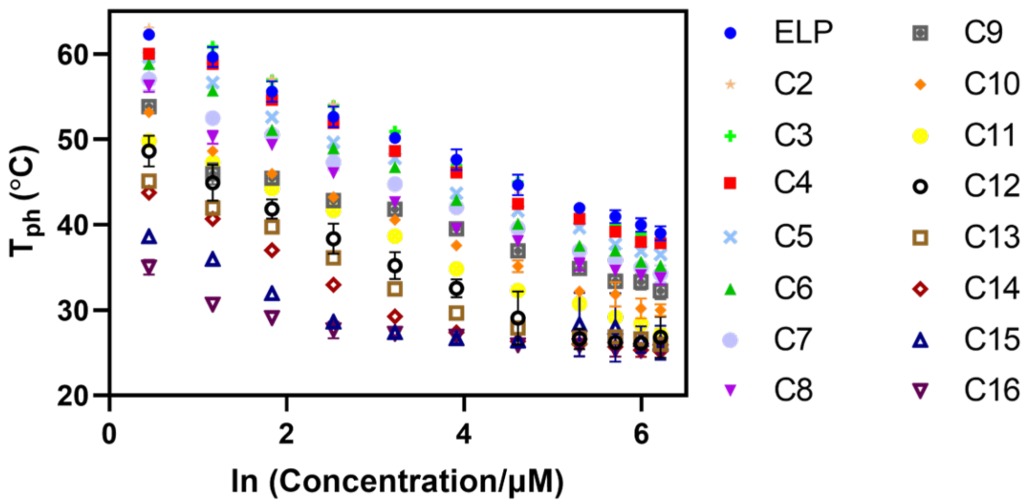

Figure S7. A partial temperature-composition phase diagram of various constructs in this study. For lipids shorter than 12 carbons, the T_{ph} maintains a consistent linear relationship with the logarithm of protein concentration, typical of unmodified ELPs. Contrarily, For C12-C16, the concentration dependence of the T_{ph} diverged from a single linear relationship and instead displayed two-segment behavior.

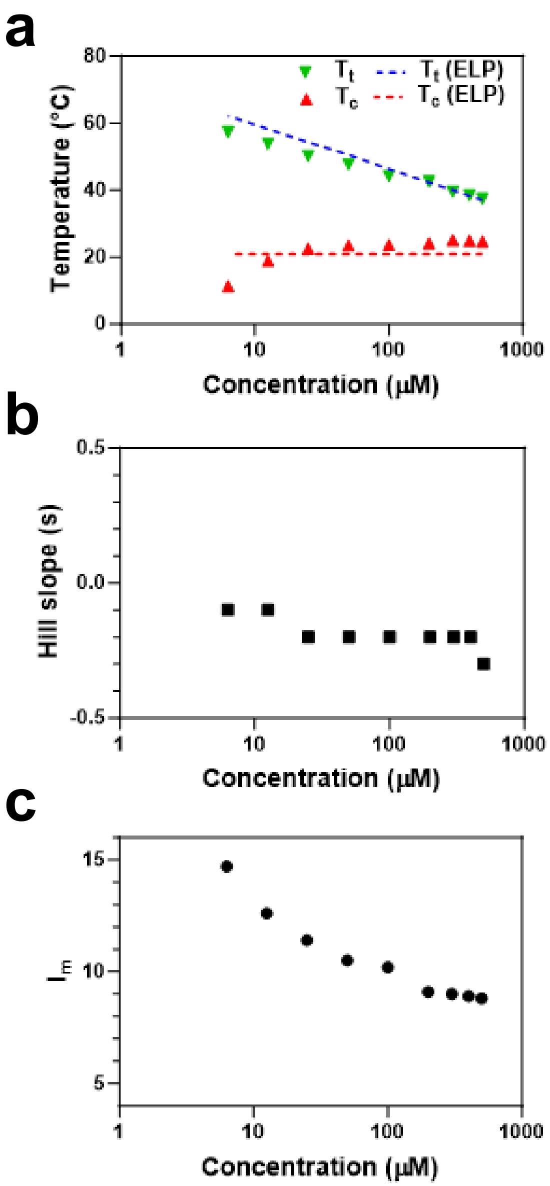

Figure S8. Concentraiton-dependence of the Curve fitting parameters correlating T_{ph} to lipid length. (a) The plot displays the T_t and T_c parameters, which are obtained from the 4PL (four-parameter logistic) model and indicated by green and red symbols, respectively. These parameters are compared with the predicted transition temperature of an unmodified ELP with a similar guest residue at two limits: 1) at similar concentration and length, as indicated by the blue dashed line; 2) at the high molecular weight limit, based on the model established by McDaniel et al.[3] **(b)** The hill slope(*s*) was approximately -0.2, although it showed a slightly negative correlation with the concentration. **(c)** The sigmoid's midpoint (l_m) was ~ 9–10 for concentrations higher than CMC (Figure S10).

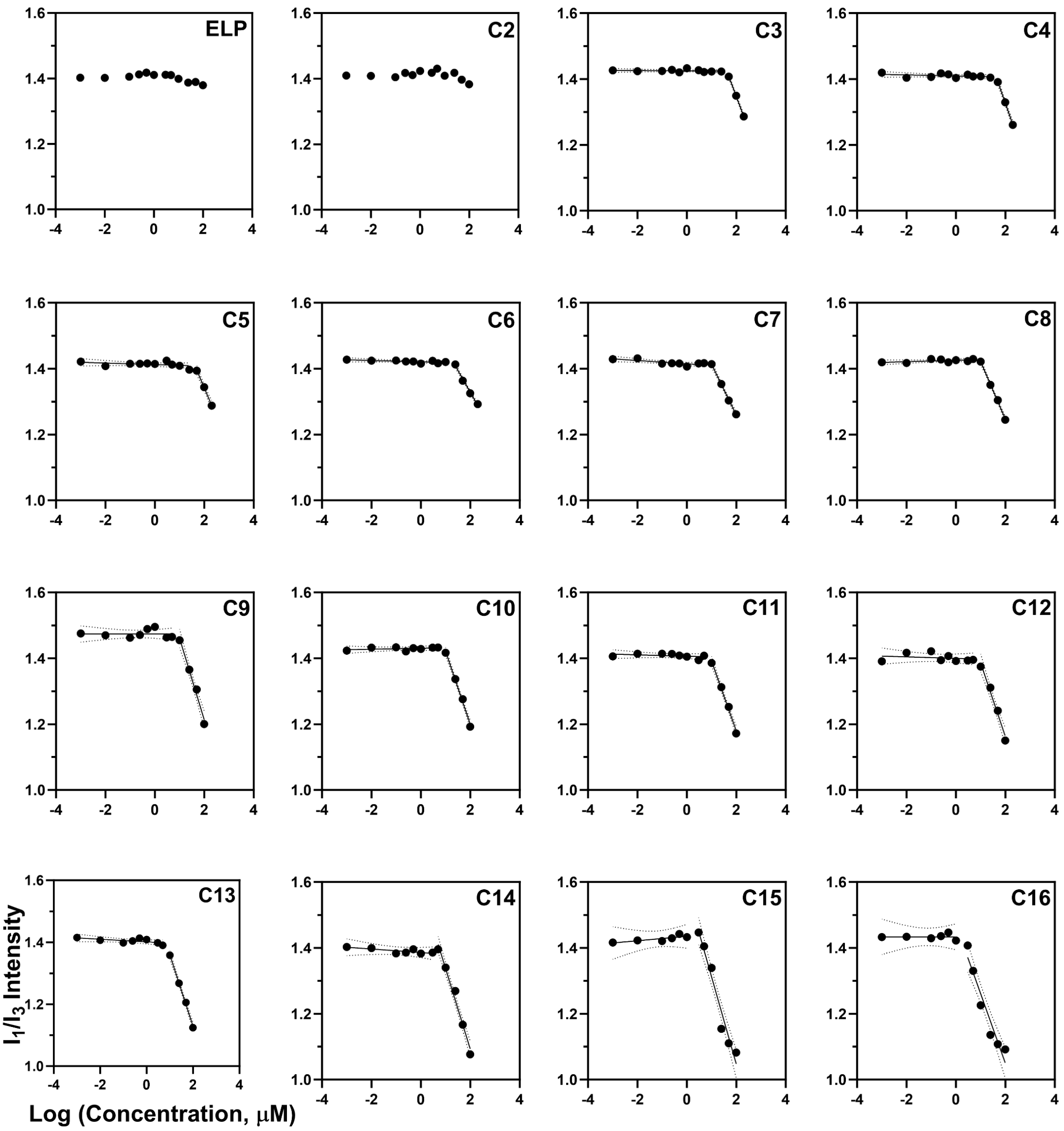

Figure S9. Pyrene assay results. I_1/I_3 intensities are displayed on a semi-log plot and fitted to a linear regression model. The critical micelle concentration (CMC) was determined from the intersection of two lines. The dotted line denotes the 95% confidence interval.

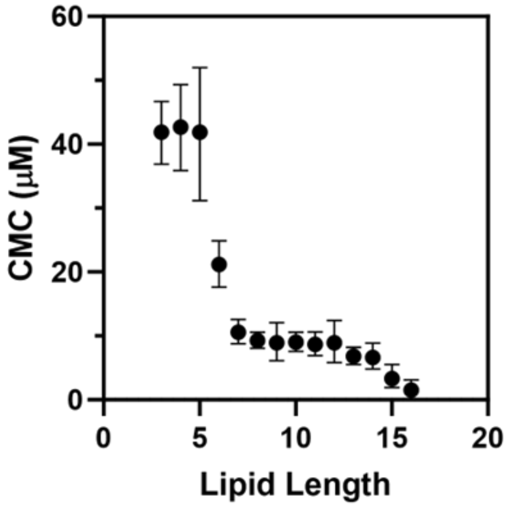

Figure S10. The variation of the critical micelle concentration (CMC) as a function of lipid length. The error bar represents the 95% confidence intervals of the intersections of the lines plotted in Figure S9. Specifically, neither the unmodified ELP nor C2 exhibited any measurable CMC up to 200 μM. However, for the remaining constructs, the CMC decreased from 40 to 3 μM as the lipid length increased from C3 to C16, consistent with prior reports.[4]

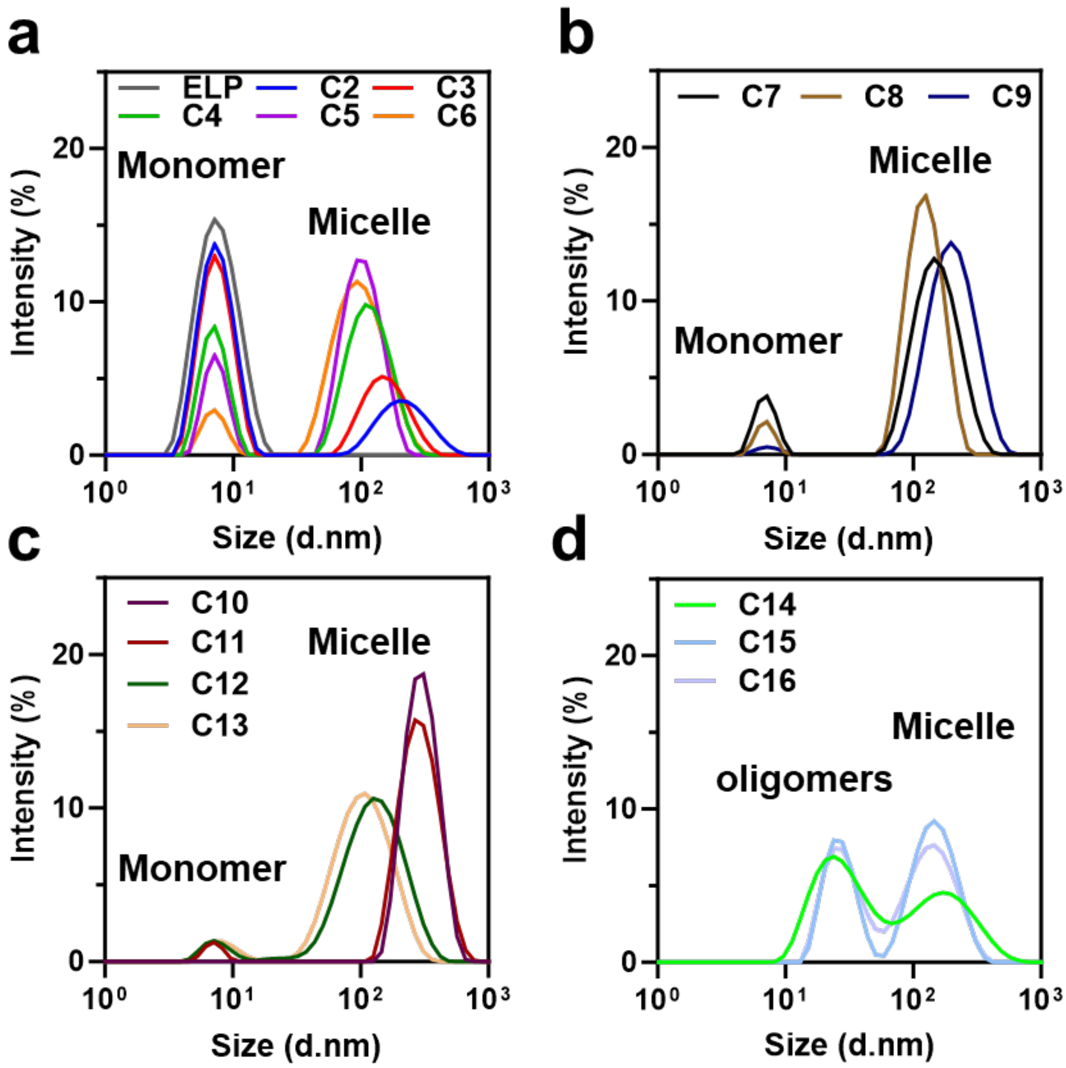

Figure S11. Dynamic light scattering analysis of FAMEs in solution. The protein concentration was maintained at 100 μM in PBS. To facilitate comparison, data are presented across multiple panels. **(a)** Unmodified ELP primarily existed in unimeric form, whereas FAMEs modified with lipids shorter than 6 carbon atoms displayed a mixture of monomer and micelles at this concentration. **(b)** For lipid lengths greater than 7 carbons (i.e., MCFA), there is a predominance of larger micelles, which is corroborated by SEC (Figure S12). **(c,d)** This trend persists with further increases in lipid length. Notably, for l≥ 14, the monomer peak is replaced by a slightly larger peak, corresponding to the oligomeric species.

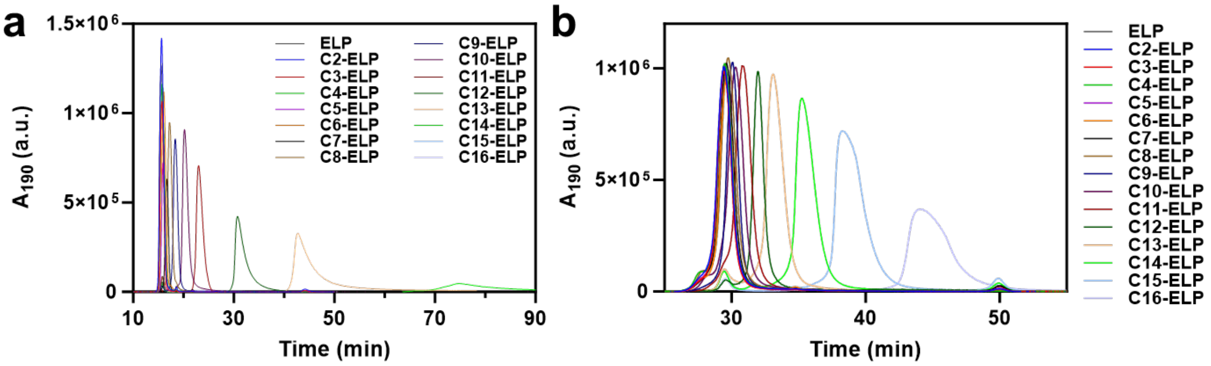

	Unimer	Micelle
**Construct**	**Retention Time (AUC%)**	**Retention Time (AUC%)**
**ELP**	**15.56 (100)**	
**C2**	**15.65 (100)**	
**C3**	**15.73 (100)**	
**C4**	**15.77 (100)**	
**C5**	**15.96 (100)**	
**C6**		**16.05 (100)**
**C7**		**16.69 (100)**
**C8**	**15.76 (5.3)**	**17.26 (94.7)**
**C9**	**15.75 (2.5)**	**18.37 (97.5)**
**C10**	**15.82 (4.6)**	**20.21 (95.4)**
**C11**	**15.56 (2.5)**	**23.00 (97.5)**
**C12**	**15.75 (3.7)**	**30.74 (96.3)**
**C13**	**15.81 (4.5)**	**42.72 (95.5)**
**C14**	**15.83 (4.1)**	**74.93 (95.9)**

Figure S12. SEC chromatograms for various constructs in this study. (a) Chromatograms obtained using (a) OHpak SB-804 HQ column (molecular weight range 5,000 – 400,000 Da) or (b) PROTEIN KW-804 column (molecular weight range 30,000 – 4,000,000 Da). The SEC data confirms that constructs with lipid lengths of 6 or greater self-assemble into micelles. The protein concentration was 50 μM in PBS. Despite different molecular weight cut-offs, both columns exhibited similar trends in relative elution time and sample composition. However, samples with lipid lengths (l) of 14 or greater displayed significantly prolonged elution times in the OHpak columns, likely due to non-specific interactions with the column packing materials.

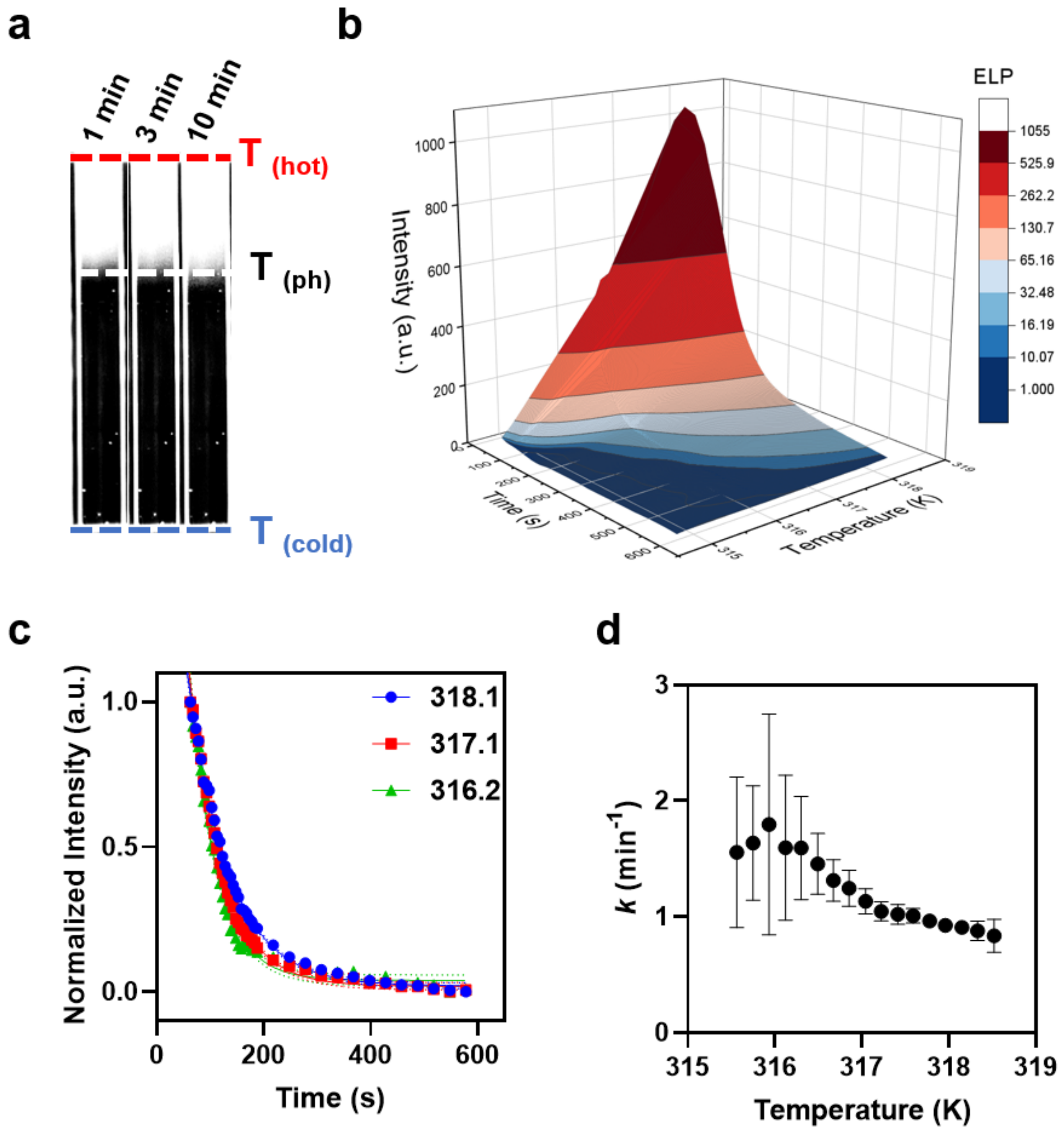

Figure S13. Kinetic analysis of ATPS formation in ELP. (a) Dark-field images of microfluidic channel placed on linear temperature gradient at t = 1, 3, and 10 min. **(b)** 3D plot of the scattering intensity as a function of time and temperature. **(c)** Representative curve fits of the intensity decay to a single-exponential decay function at the three temperatures. **(d)** Rate constant as a function of temperature. Error bars represent the standard deviations of three measurements. Concentration is 10 mg/mL.

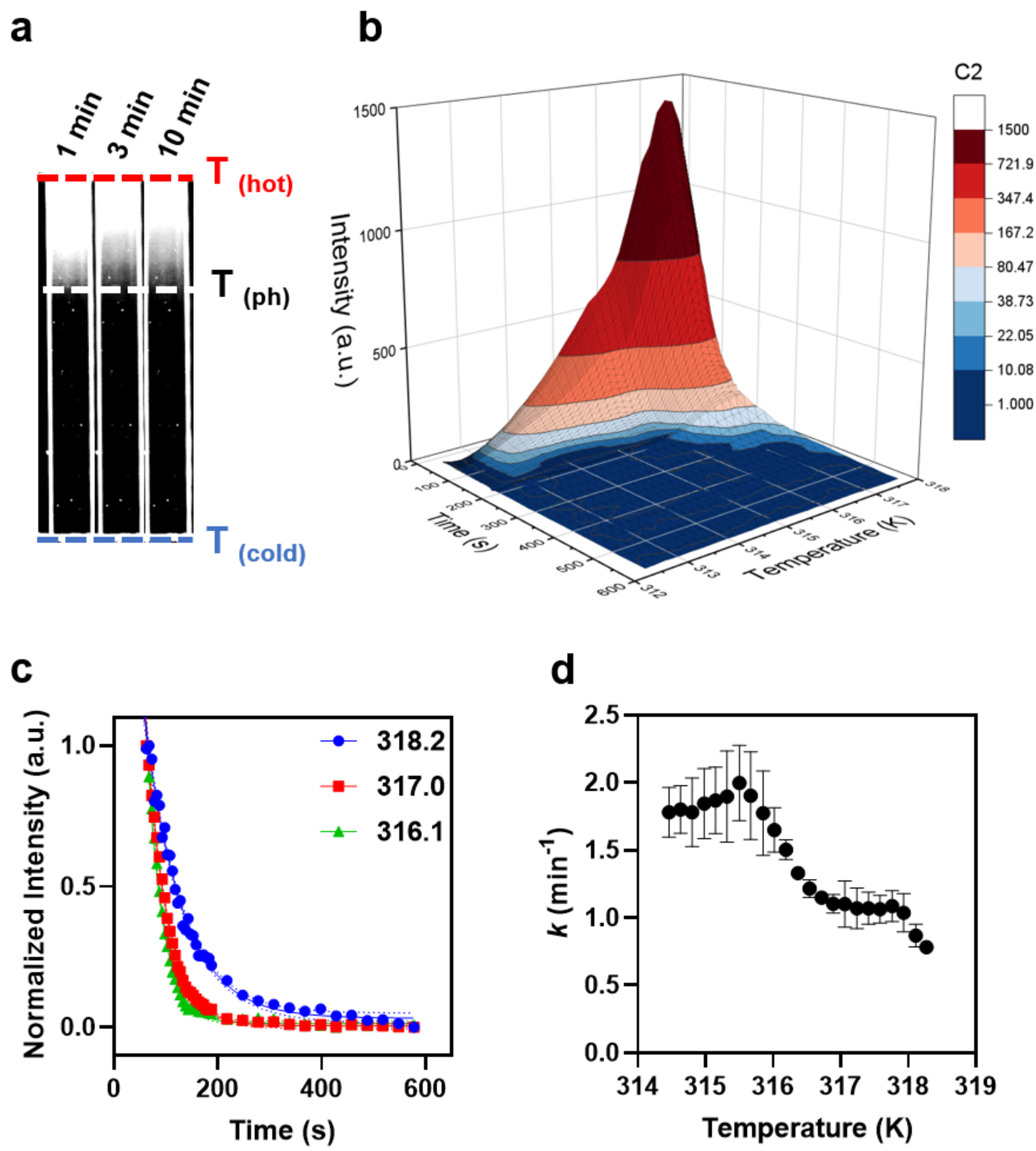

Figure S14. Kinetic analysis of ATPS formation in C2-ELP. (a) Dark-field images of microfluidic channel placed on linear temperature gradient at t = 1, 3, and 10 min. **(b)** 3D plot of the scattering intensity as a function of time and temperature. **(c)** Representative curve fits of the intensity decay to a single-exponential decay function at the three temperatures. **(d)** Rate constant as a function of temperature. Error bars represent the standard deviations of three measurements. Concentration is 10 mg/mL.

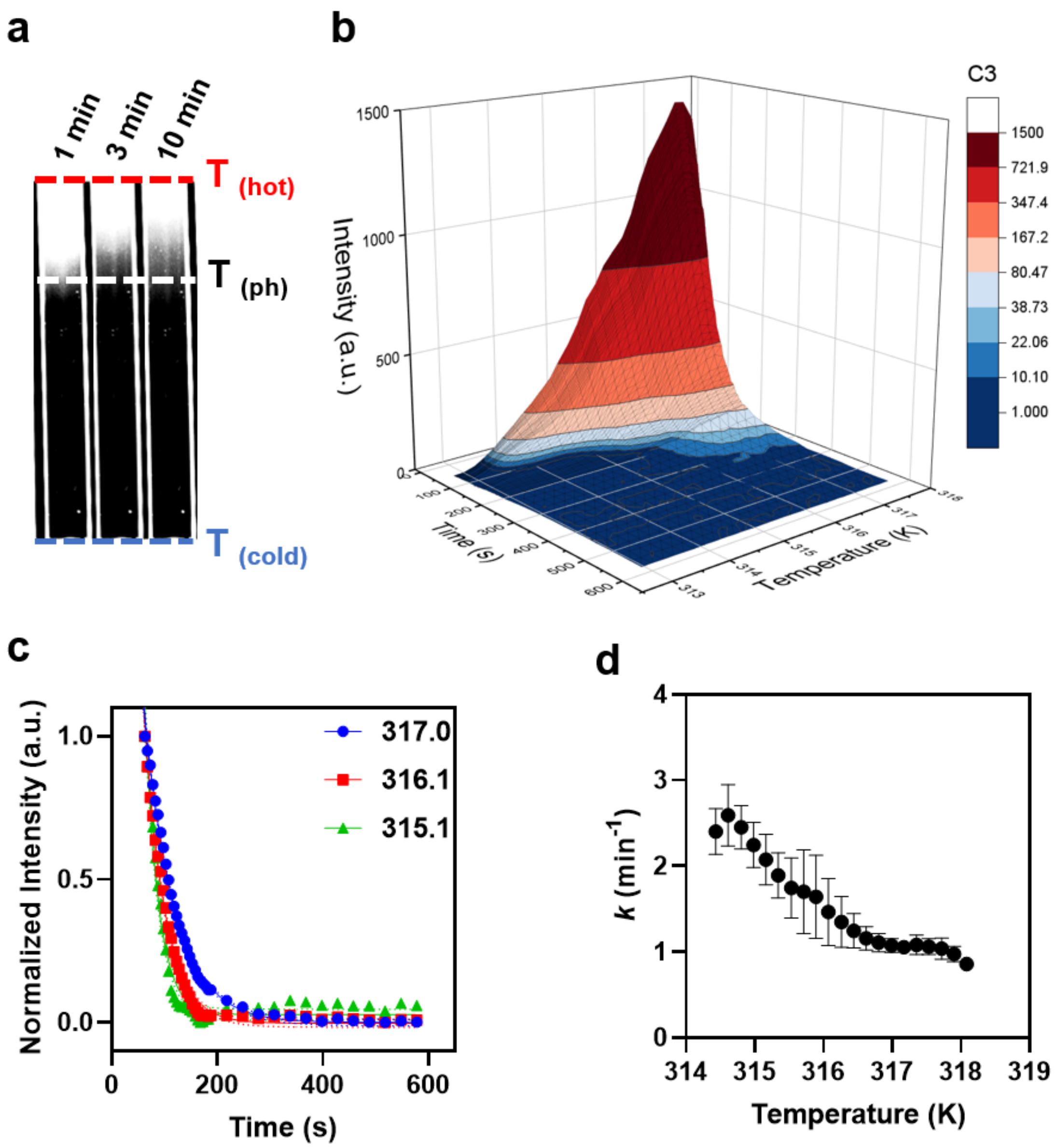

Figure S15. Kinetic analysis of ATPS formation in C3-ELP. (a) Dark-field images of the microfluidic channel placed on a linear temperature gradient at t = 1, 3, and 10 min. **(b)** 3D plot of the scattering intensity as a function of time and temperature. **(c)** Representative curve fits of the intensity decay to a single-exponential decay function at the three temperatures. **(d)** Rate constant as a function of temperature. Error bars represent the standard deviations of three measurements. Concentration is 10 mg/mL.

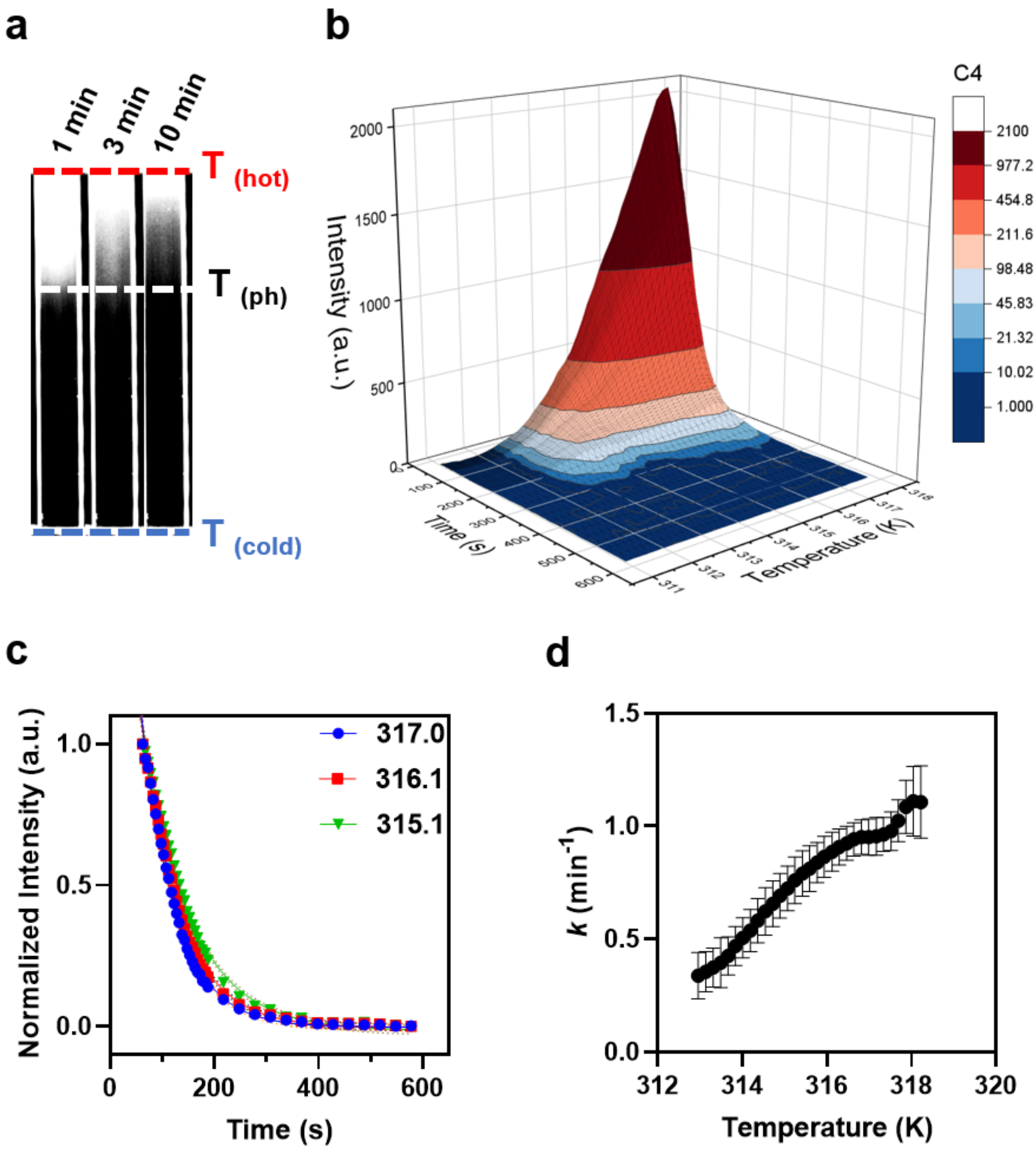

Figure S16. Kinetic analysis of ATPS formation in C4-ELP. (a) Dark-field images of the microfluidic channel placed on a linear temperature gradient at t = 1, 3, and 10 min. **(b)** 3D plot of the scattering intensity as a function of time and temperature. **(c)** Representative curve fits of the intensity decay to a single-exponential decay function at the three temperatures. **(d)** Rate constant as a function of temperature. Error bars represent the standard deviations of three measurements. Concentration is 10 mg/mL.

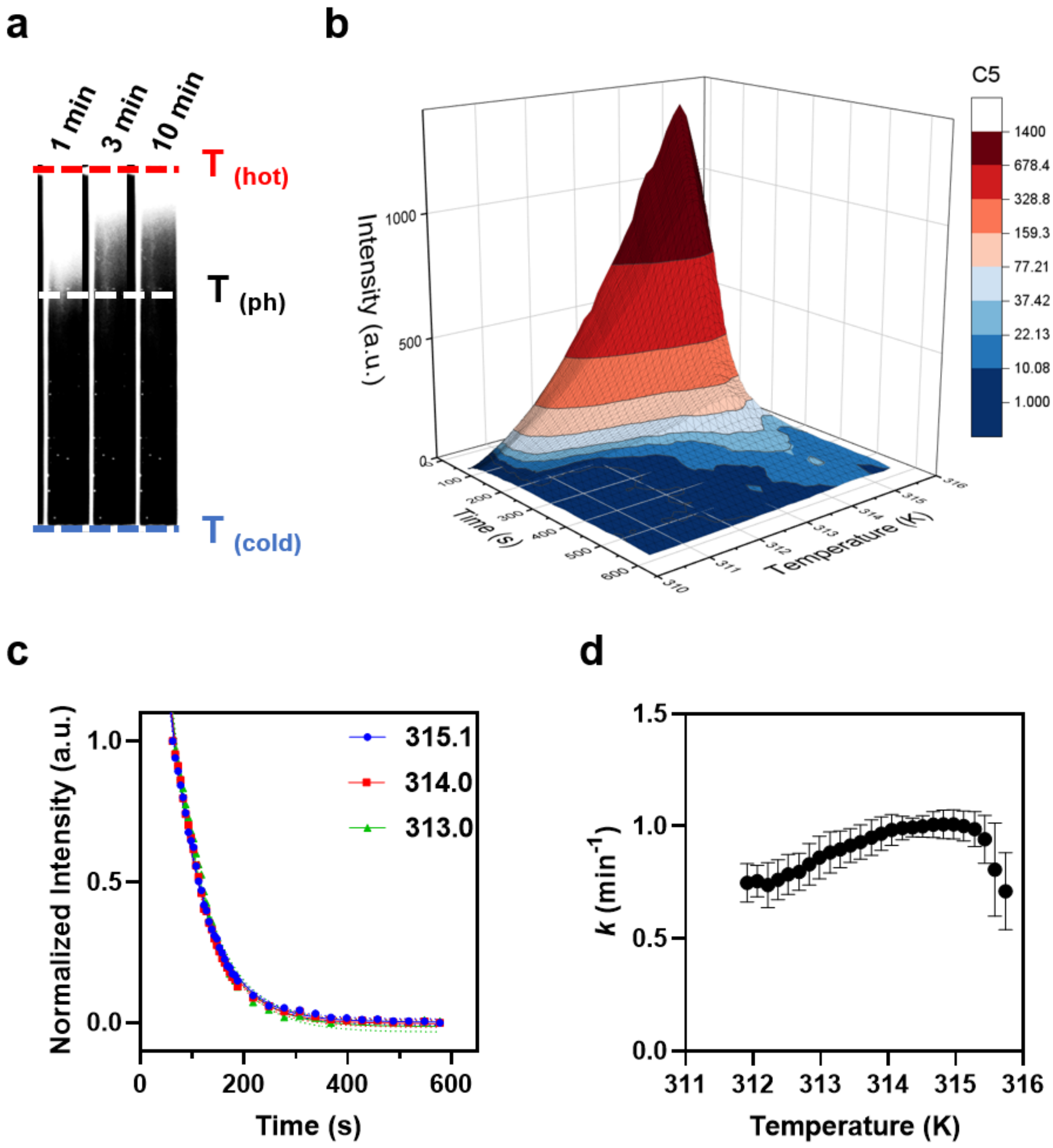

Figure S17. Kinetic analysis of ATPS formation in C5-ELP. (a) Dark-field images of microfluidic channel placed on linear temperature gradient at t = 1, 3, and 10 min. **(b)** 3D plot of the scattering intensity as a function of time and temperature. **(c)** Representative curve fits of intensity decay to a single-exponential decay function at three temperatures. **(d)** Rate constant as a function of temperature. Error bars represent the standard deviations of three measurements. Concentration is 10 mg/mL.

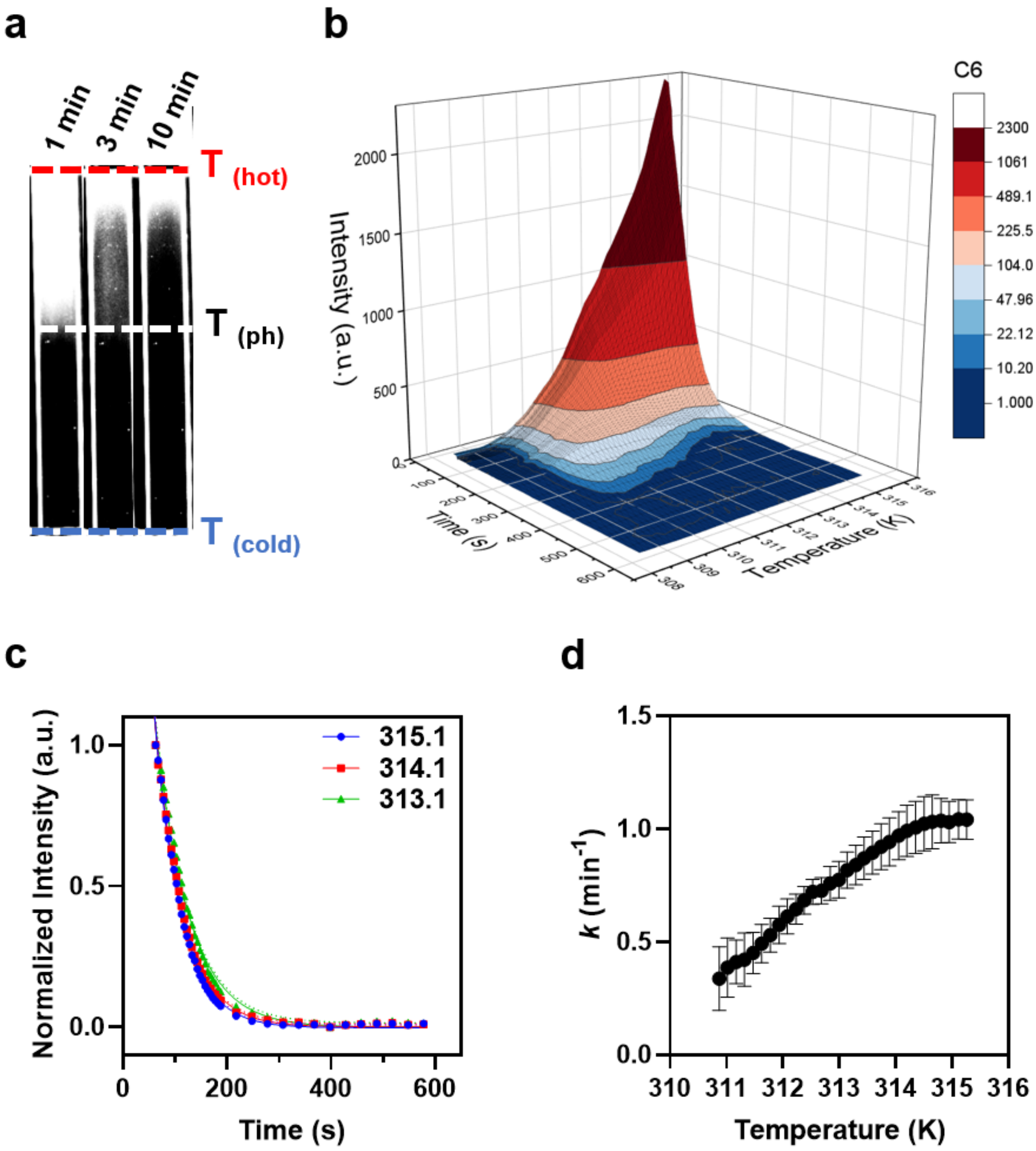

Figure S18. Kinetic analysis of ATPS formation in C6-ELP. (a) Dark-field images of microfluidic channel placed on linear temperature gradient at t = 1, 3, and 10 min. **(b)** 3D plot of the scattering intensity as a function of time and temperature. **(c)** Representative curve fits of intensity decay to a single-exponential decay function at three temperatures. **(d)** Rate constant as a function of temperature. Error bars represent the standard deviations of three measurements. Concentration is 10 mg/mL.

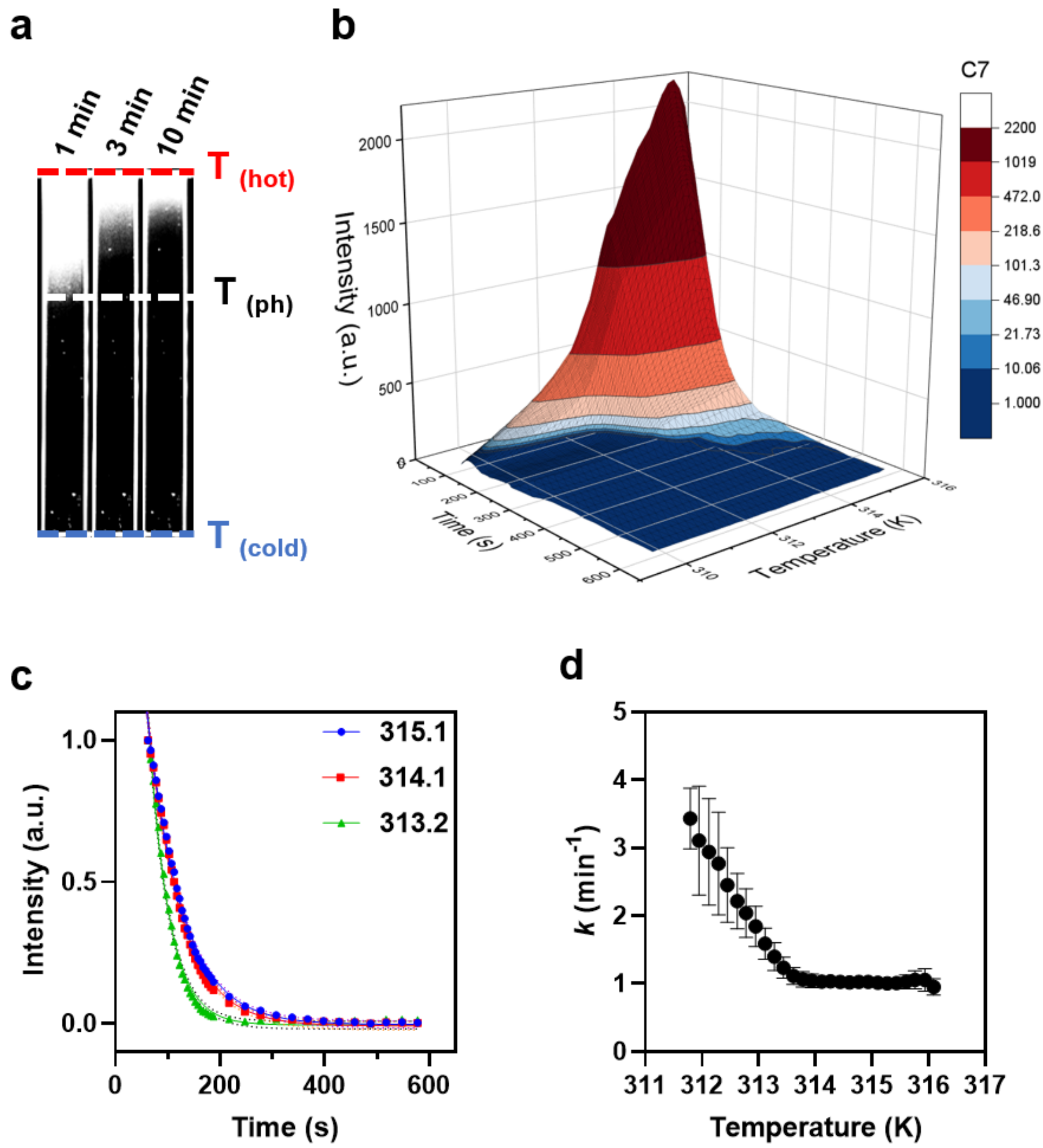

Figure S19. Kinetic analysis of ATPS formation in C7-ELP. (a) Dark-field images of microfluidic channel placed on linear temperature gradient at t = 1, 3, and 10 min. **(b)** 3D plot of the scattering intensity as a function of time and temperature. **(c)** Representative curve fits of intensity decay to a single-exponential decay function at three temperatures. **(d)** Rate constant as a function of temperature. Error bars represent the standard deviations of three measurements. Concentration is 10 mg/mL.

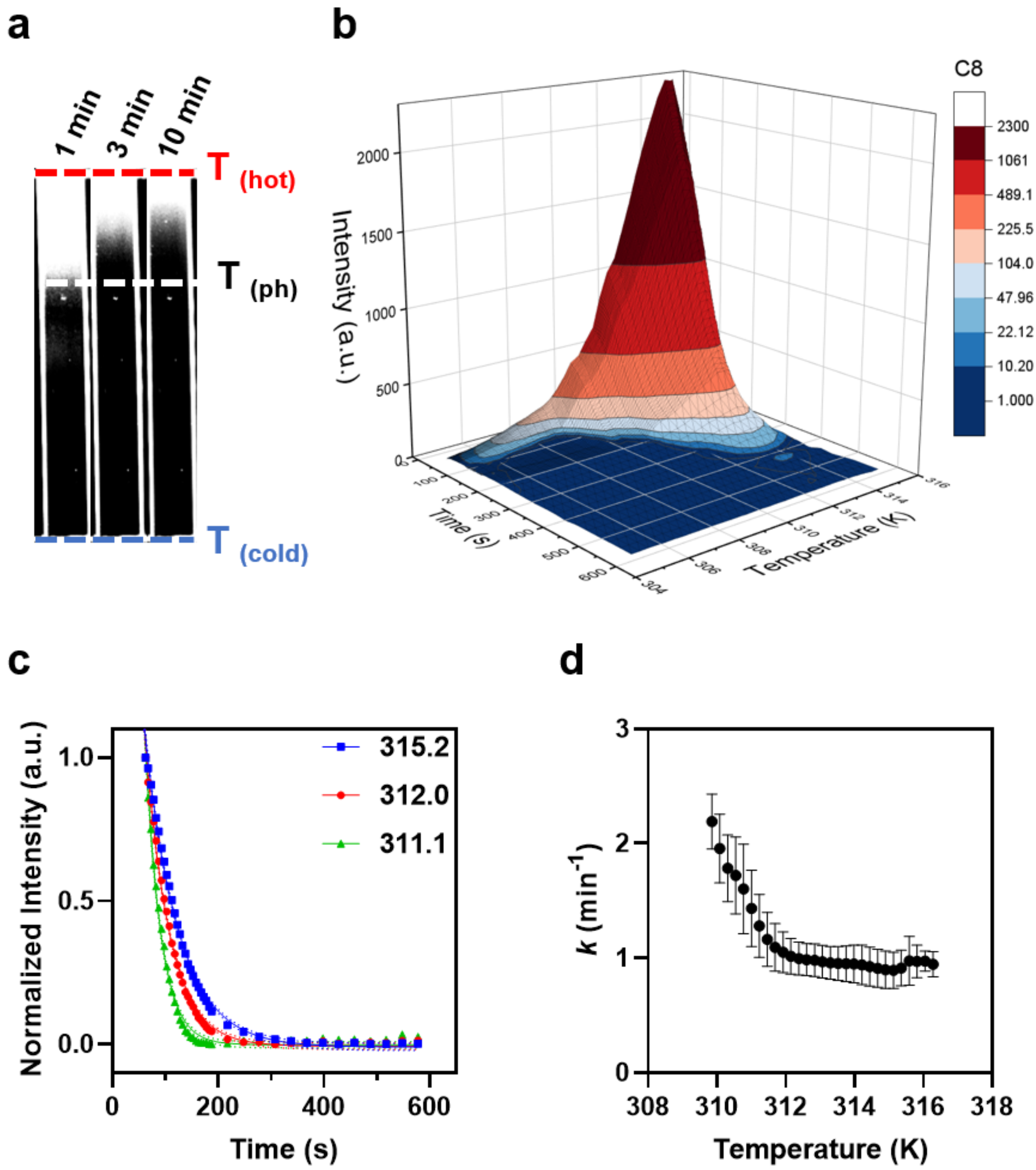

Figure S20. Kinetic analysis of ATPS formation in C8-ELP. (a) Dark-field images of microfluidic channel placed on linear temperature gradient at t = 1, 3, and 10 min. **(b)** 3D plot of the scattering intensity as a function of time and temperature. **(c)** Representative curve fits of intensity decay to a single-exponential decay function at three temperatures. **(d)** Rate constant as a function of temperature. Error bars represent the standard deviations of three measurements. Concentration is 10 mg/mL.

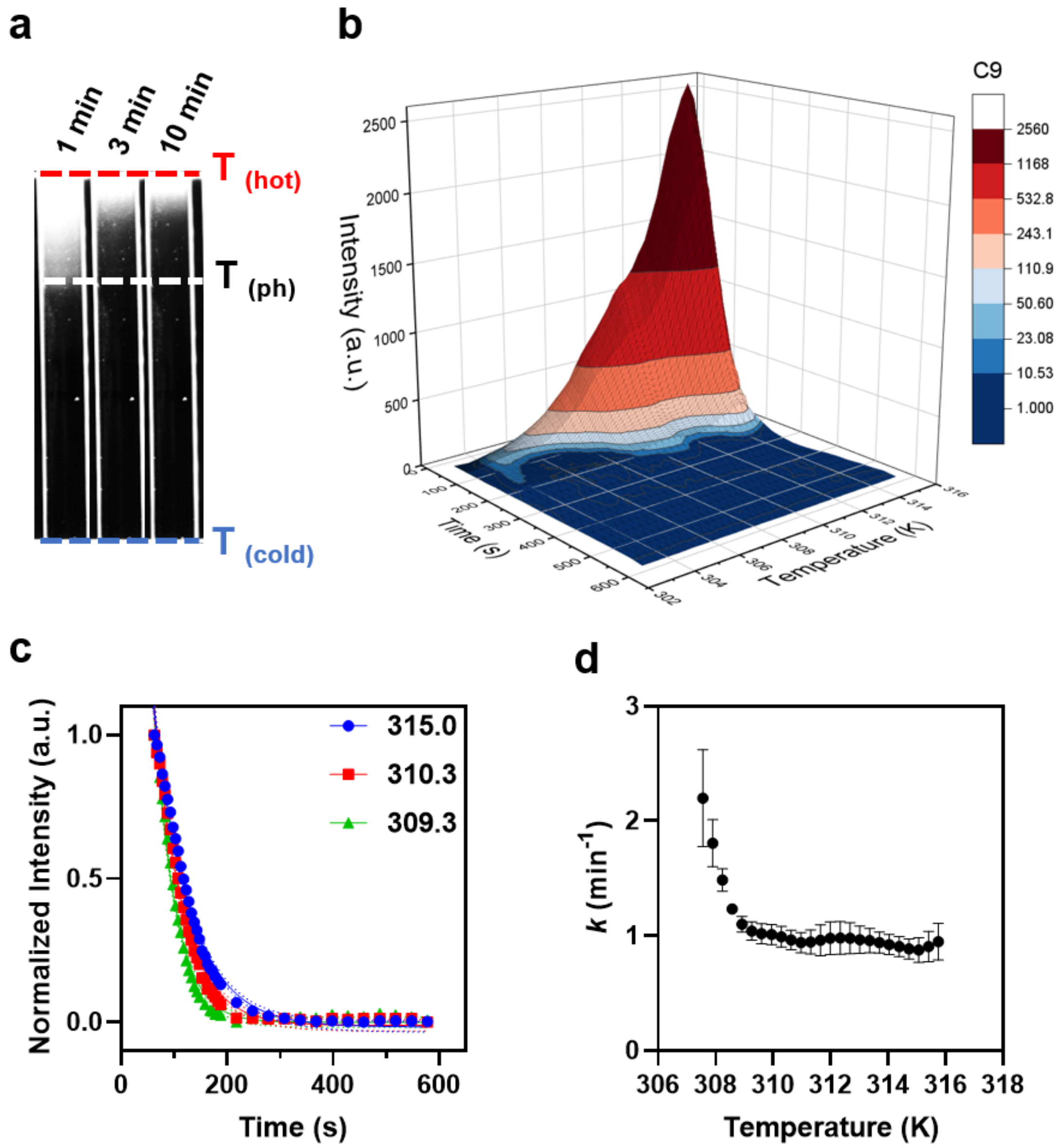

Figure S21. Kinetic analysis of ATPS formation in C9-ELP. (a) Dark-field images of microfluidic channel placed on linear temperature gradient at t = 1, 3, and 10 min. **(b)** 3D plot of the scattering intensity as a function of time and temperature. **(c)** Representative curve fits of intensity decay to a single-exponential decay function at three temperatures. **(d)** Rate constant as a function of temperature. Error bars represent the standard deviations of three measurements. Concentration is 10 mg/mL.

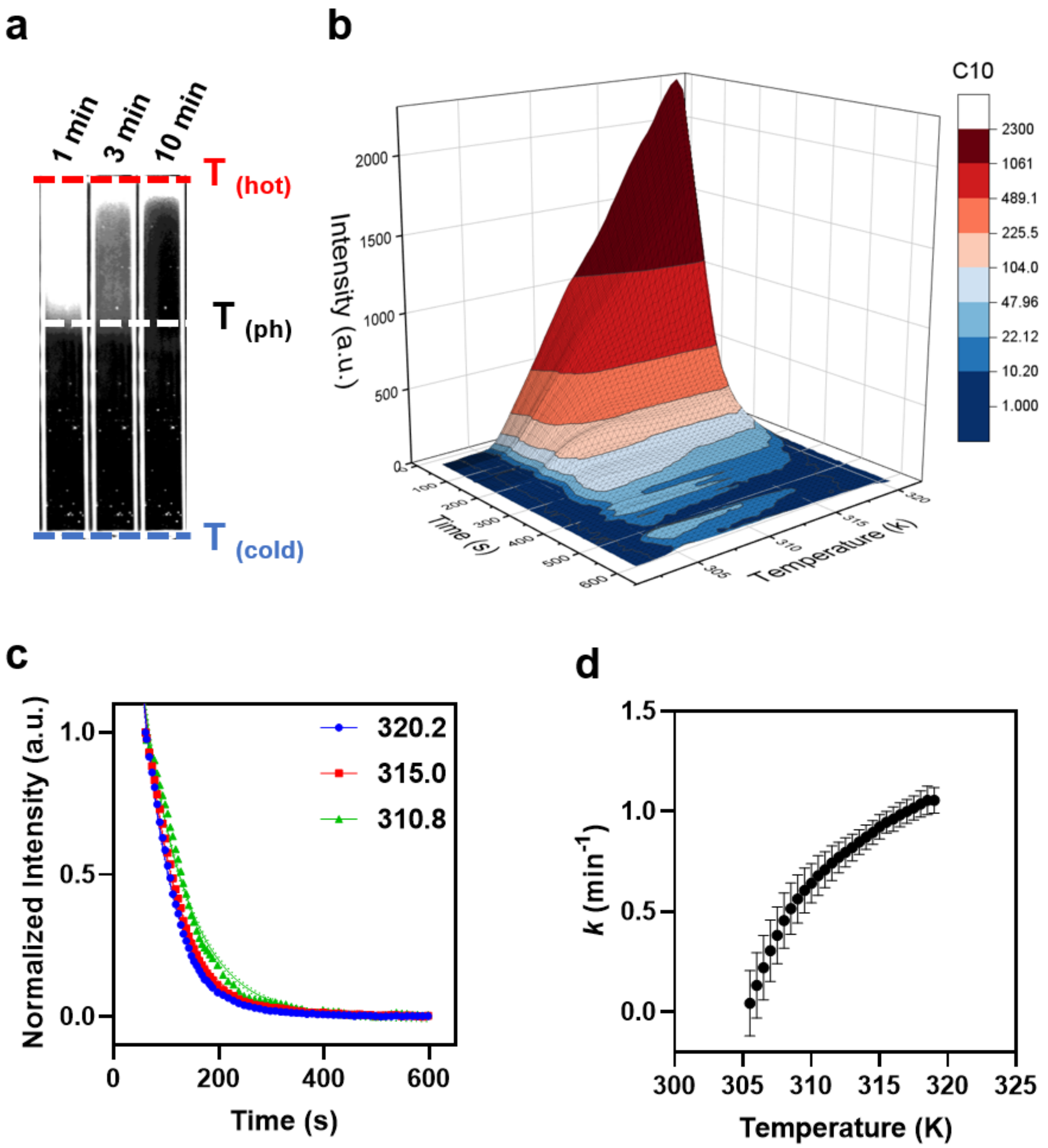

Figure S22. Kinetic analysis of ATPS formation in C10-ELP. (a) Dark-field images of microfluidic channel placed on linear temperature gradient at t = 1, 3, and 10 min. **(b)** 3D plot of the scattering intensity as a function of time and temperature. **(c)** Representative curve fits of intensity decay to a single-exponential decay function at three temperatures. **(d)** Rate constant as a function of temperature. Error bars represent the standard deviations of six measurements. Concentration is 10 mg/mL.

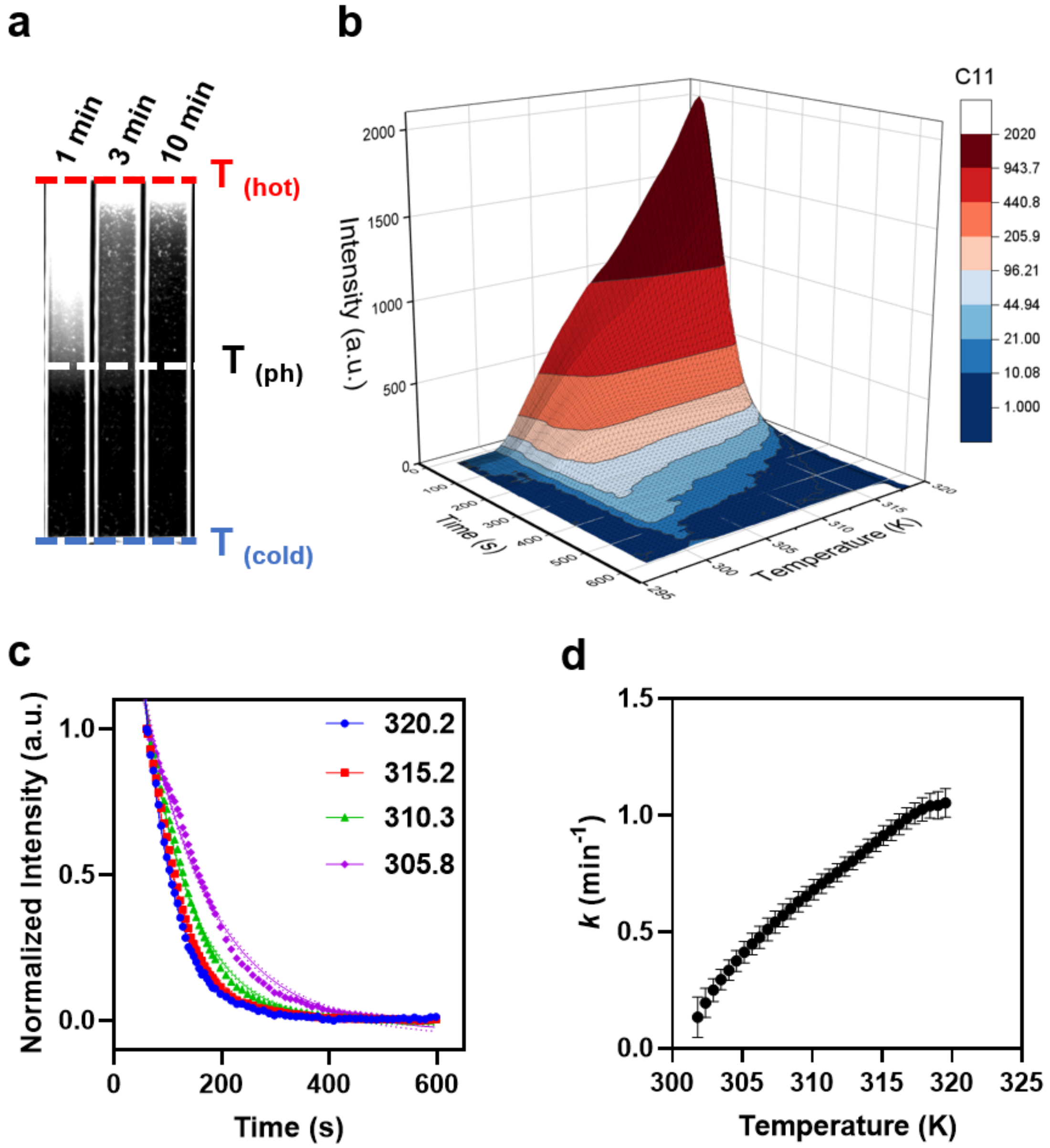

Figure S23. Kinetic analysis of ATPS formation in C11-ELP. (a) Dark-field images of microfluidic channel placed on linear temperature gradient at t = 1, 3, and 10 min. **(b)** 3D plot of the scattering intensity as a function of time and temperature. **(c)** Representative curve fits of the intensity decay to a single-exponential decay function at the four temperatures. **(d)** Rate constant as a function of temperature. Error bars represent the standard deviations of six measurements. Concentration is 10 mg/mL.

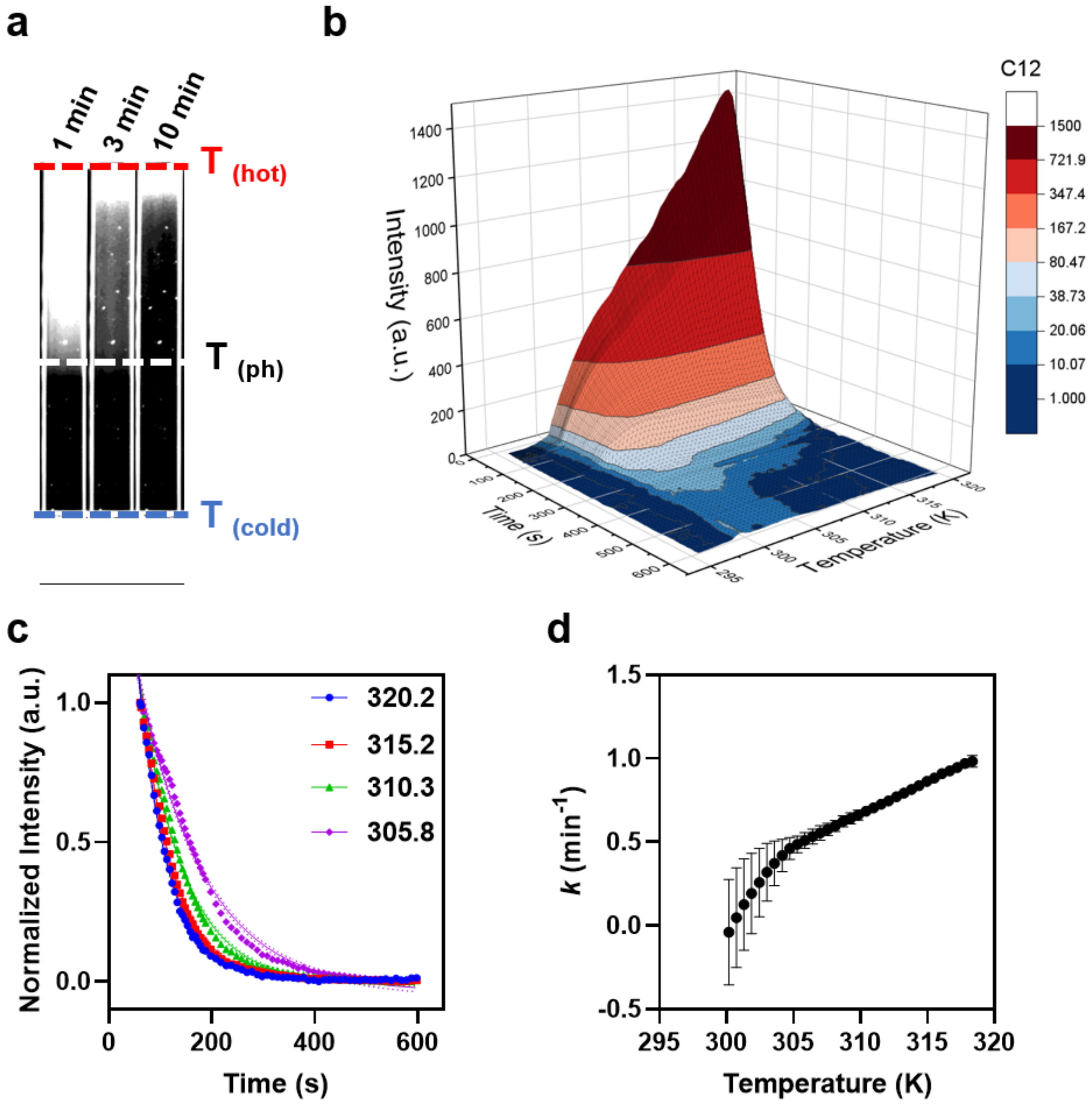

Figure S24. Kinetic analysis of ATPS formation in C12-ELP. (a) Dark-field images of microfluidic channel placed on linear temperature gradient at t = 1, 3, and 10 min. **(b)** 3D plot of the scattering intensity as a function of time and temperature. **(c)** Representative curve fits of the intensity decay to a single-exponential decay function at the four temperatures. (d) Rate constant as a function of temperature. Error bars represent the standard deviations of six measurements. Concentration is 10 mg/mL.

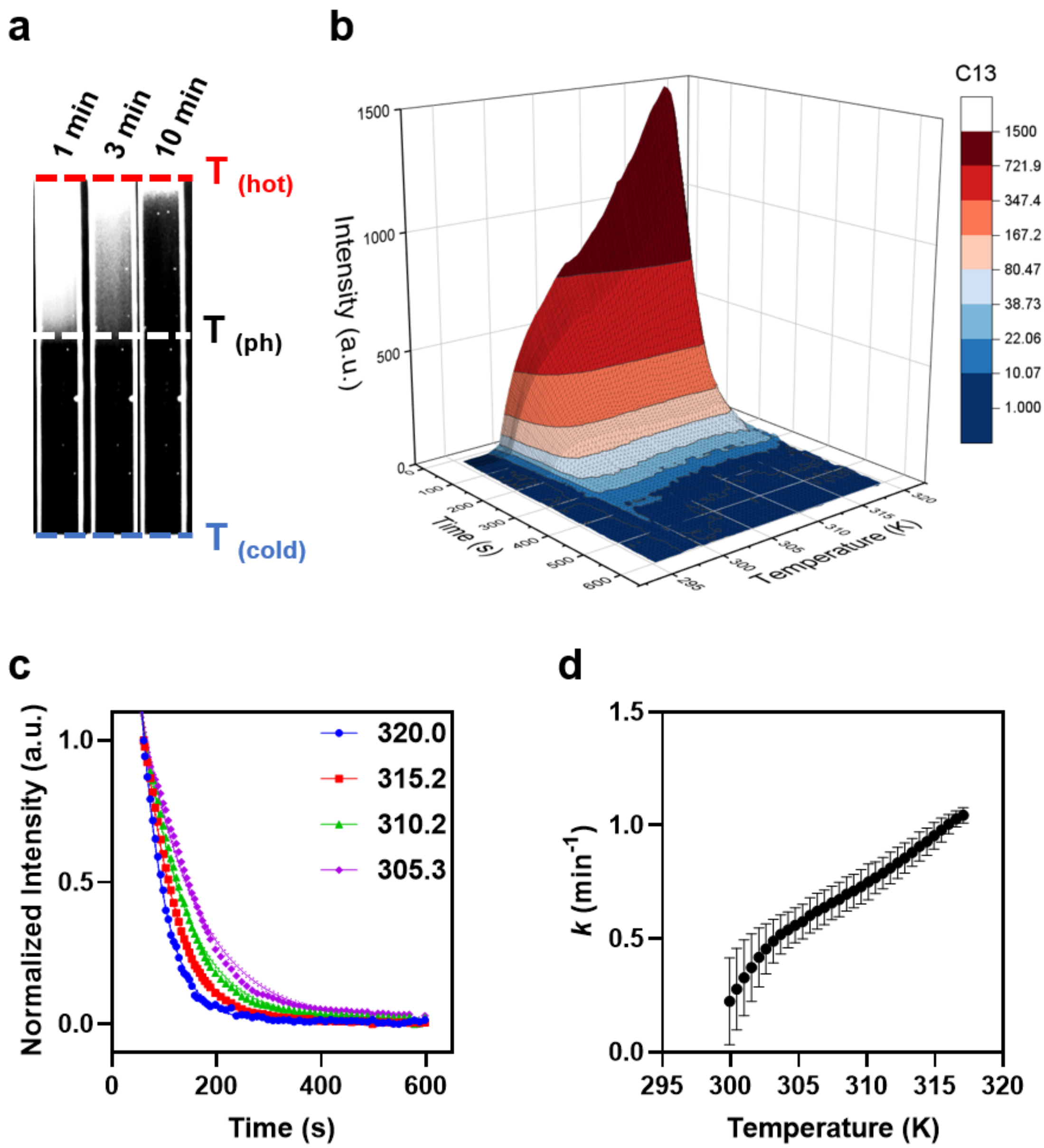

Figure S25. Kinetic analysis of ATPS formation in C13-ELP. (a) Dark-field images of microfluidic channel placed on linear temperature gradient at t = 1, 3, and 10 min. **(b)** 3D plot of the scattering intensity as a function of time and temperature. **(c)** Representative curve fits of the intensity decay to a single-exponential decay function at the four temperatures. **(d)** Rate constant as a function of temperature. Error bars represent the standard deviations of six measurements. Concentration is 10 mg/mL.

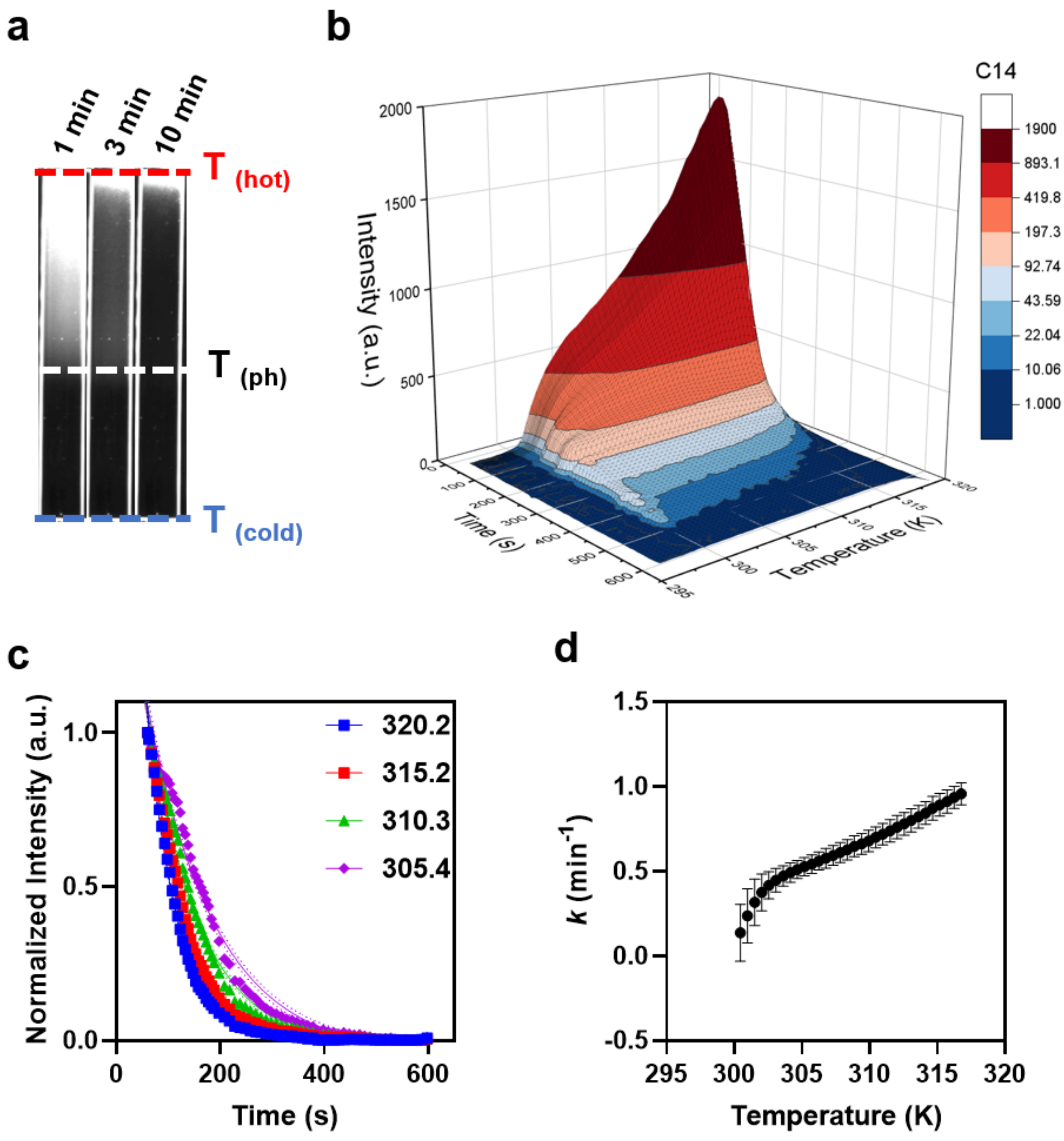

Figure S26. Kinetic analysis of ATPS formation in C14-ELP. (a) Dark-field images of microfluidic channel placed on linear temperature gradient at t = 1, 3, and 10 min. **(b)** 3D plot of the scattering intensity as a function of time and temperature. **(c)** Representative curve fits of the intensity decay to a single-exponential decay function at the four temperatures. **(d)** Rate constant as a function of temperature. Error bars represent the standard deviations of six measurements. Concentration is 10 mg/mL.

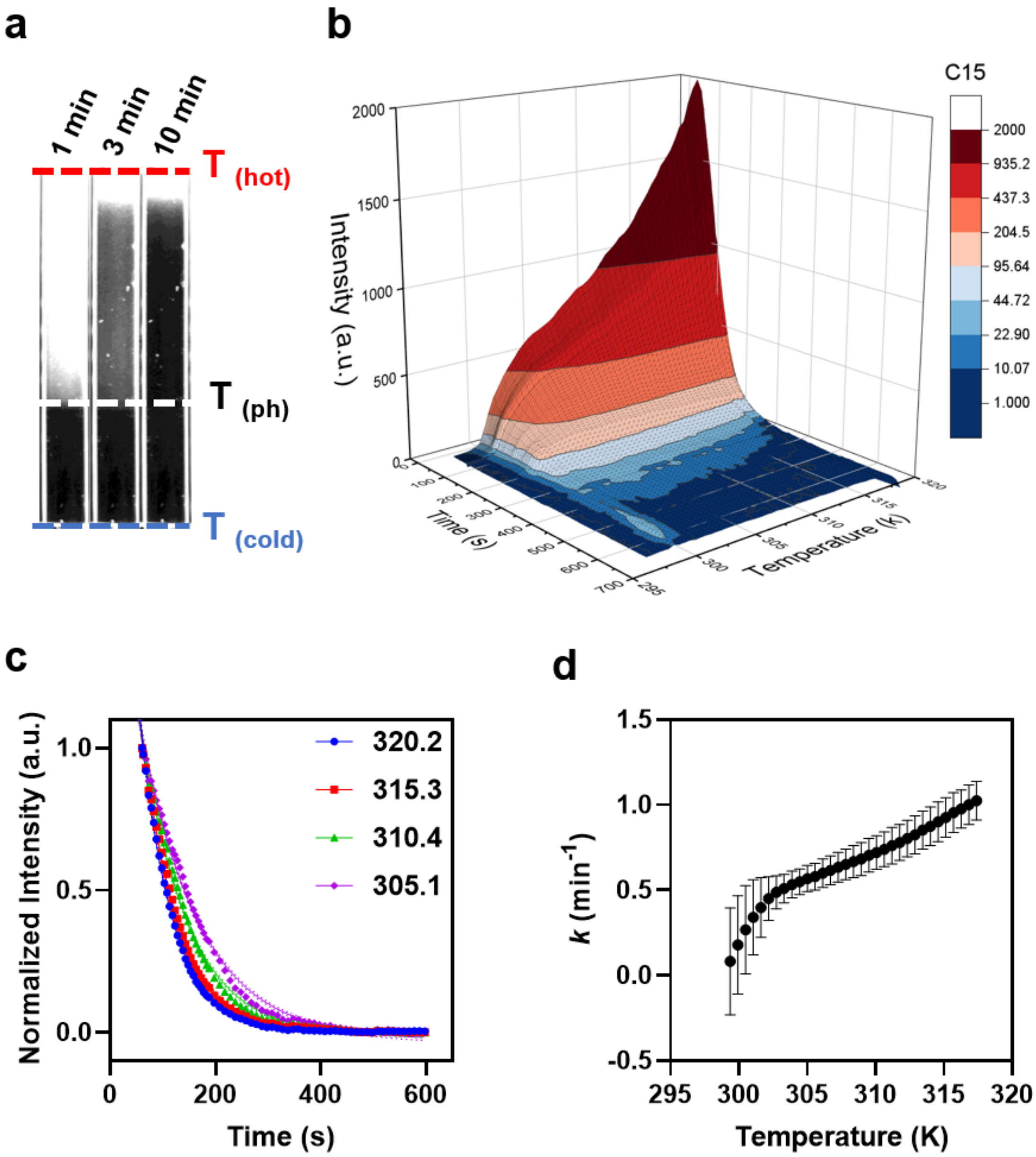

Figure S27. Kinetic analysis of ATPS formation in C15-ELP. (a) Dark-field images of microfluidic channel placed on linear temperature gradient at t = 1, 3, and 10 min. **(b)** 3D plot of the scattering intensity as a function of time and temperature. **(c)** Representative curve fits of the intensity decay to a single-exponential decay function at the four temperatures. **(d)** Rate constant as a function of temperature. Error bars represent the standard deviations of six measurements. Concentration is 10 mg/mL.

4. References

(1) Ovčačíková, M.; Lísa, M.; Cífková, E.; Holčapek, M. Retention behavior of lipids in reversed-phase ultrahigh-performance liquid chromatography–electrospray ionization mass spectrometry. *J. Chromatogr. A* **2016**, *1450*, 76-85.

(2) Lienqueo, M. E.; Mahn, A.; Vásquez, L.; Asenjo, J. A. Methodology for predicting the separation of proteins by hydrophobic interaction chromatography and its application to a cell extract. *J. Chromatogr. A* **2003**, *1009* (1-2), 189-196.

(3) McDaniel, J. R.; Radford, D. C.; Chilkoti, A. A Unified Model for De Novo Design of Elastin-like Polypeptides with Tunable Inverse Transition Temperatures. *Biomacromolecules* **2013**, *14* (8), 2866-2872. DOI: 10.1021/bm4007166.

(4) Zhang, T.; Peruch, F.; Weber, A.; Bathany, K.; Fauquignon, M.; Mutschler, A.; Schatz, C.; Garbay, B. Solution behavior and encapsulation properties of fatty acid–elastin-like polypeptide conjugates. *RSC Adv.* **2023**, *13* (3), 2190-2201. DOI: 10.1039/D2RA06603C.
